# Hydrogen release from a single water molecule on V_*n*_^+^ (3 ≤ *n* ≤ 30)

**DOI:** 10.1038/s42004-020-00396-9

**Published:** 2020-10-30

**Authors:** Hanyu Zhang, Haiming Wu, Yuhan Jia, Baoqi Yin, Lijun Geng, Zhixun Luo, Klavs Hansen

**Affiliations:** 1 Beijing National Laboratory of Molecular sciences (BNLMS), State Key Laboratory for Structural Chemistry of Unstable and Stable Species, Institute of Chemistry, Chinese Academy of Sciences, Beijing, 100190 P.R. China; 2 University of Chinese Academy of Sciences, Beijing, 100049 P.R. China; 3 Joint Centre for Quantum Studies and Department of Physics, School of Science, Tianjin University, Tianjin, P.R. China

**Keywords:** Catalytic mechanisms, Heterogeneous catalysis, Energy, Reaction kinetics and dynamics

## Abstract

Water and its interactions with metals are closely bound up with human life, and the reactivity of metal clusters with water is of fundamental importance for the understanding of hydrogen generation. Here a prominent hydrogen evolution reaction (HER) of single water molecule on vanadium clusters V_*n*_^+^ (3 ≤ *n* ≤ 30) is observed in the reaction of cationic vanadium clusters with water at room temperature. The combined experimental and theoretical studies reveal that the wagging vibrations of a V-OH group give rise to readily formed V-O-V intermediate states on V_*n*_^+^ (*n* ≥ 3) clusters and allow the terminal hydrogen to interact with an adsorbed hydrogen atom, enabling hydrogen release. The presence of three metal atoms reduces the energy barrier of the rate-determining step, giving rise to an effective production of hydrogen from single water molecules. This mechanism differs from dissociative chemisorption of multiple water molecules on aluminium cluster anions, which usually proceeds by dissociative chemisorption of at least two water molecules at multiple surface sites followed by a recombination of the adsorbed hydrogen atoms.

## Abstract (web summary)

Metal clusters are known to catalyze hydrogen evolution from water, but reaction mechanisms and reactivities are still not fully understood. Here, mass spectrometry of cationic vanadium clusters reacting with water vapor shows significant hydrogen evolution reactions for V_3_^+^, V_5_^+^ and V_9_^+^ that point to a hydrogen release mechanism from the transition metal cluster cations.

## Introduction

Generating hydrogen from water is one of the attractive research fields motivated by the quest for sources of clean energy in modern society^1,2^. Numerous strategies have been developed for hydrogen production including electrolysis^3–5^, artificial photosynthesis^6^, use of photocatalysts^7,8^, and thermal decomposition of water^9^. In the ongoing efforts devoted to exploring effective catalysts and cluster supports for water splitting^10–13^, aluminium alloy powders with unique nanoscale galvanic microstructure have been shown to produce hydrogen gas upon contact with water^14^. Also, hydrogen elimination from Al-based hydrates, e.g., [Al, 20H_2_O]^+^, were proposed in previous studies^15–17^, where the proton transfer in the water cluster network enables a migrated proton to recombine with a hydridic H at the Al^III^ cation, and a joint experimental and theoretical study illustrated the potential application of pure aluminium cluster anions for effective production of H_2_ from water^18^. In these experiments, active sites on the surface of the aluminium clusters, typically Al_16_^−^, Al_17_^−^, and Al_18_^−^, produced hydrogen from contact with water at room temperature. These reactions are initiated by dissociative chemisorption of a few water molecules at different surface sites, and continues by the Tafel reaction mechanism of recombination of adsorbed hydrogen atoms (i.e., H_ad_ + H_ad_ → H_2_)^19,20^. In a further development, the complementary-active-sites (CAS) mechanism^18^ has been established to rationalize the size-selective reactivity of Al clusters with polar molecules beyond water^21–26^ and have demonstrated the existence of competition of water versus alcohols in reactions with Al clusters^27^, have illustrated how partial atomic charges and bonding orbitals^28^ and the doping of heteroatoms affects such HER processes^29–33^, and have demonstrated the Eley–Rideal and Langmuir–Hinshelwood mechanisms in the presence of two OH-group molecules^34^.

Such advances have stimulated further studies of cluster reactivity of transition metal clusters in view of their *d*-electron activity. As an important family of functional materials, transition metal oxides show high propensity for a variety of catalytic reactions, including oxygen evolution reactions. Also, the interactions of light transition metal cations with compounds containing prototypical bonds (e.g., N–H, O–H) have attracted a great deal of attention over the past years^35–39^. For example, a study of the hydrated vanadium cations has shown that absorption of ambient blackbody radiation enables O-atom transfer from H_2_O to metal, followed by hydrogen evolution^40–42^. Also, vanadium ions in their ^3^F and other higher lying states were found to react with water^40,43^, however, for the ground-state cation (V^+^, ^5^D) which is ~1.08 eV lower in energy^44,45^, the H_2_ release suffers from a large energy barrier due to the absence of active sites. As the binding energies of V_*n*_^+^H_2_O (*n* = 2–13) are much smaller than that of V^+^H_2_O^46^, this opens the possibility that similar mechanisms can be present for clusters, which is what motivated the present studies of the HER efficiency on V_*n*_^+^ clusters.

For the experiments reported here, we have prepared mass-resolved cationic vanadium clusters (V_*n*_^+^) and have observed their reactions with water vapour in a compact flow tube reactor. The composition of the reaction products were measured with a homemade reflection time-of-flight mass spectrum (Re-TOFMS)^47^. Three series of reaction products, V_*n*_O^+^, V_*n*_O_2_^+^ and V_*n*_O_3_^+^, were identified, corresponding to successive reactions of V_*n*_^+^ with H_2_O molecules. Both the monomer and dimer, V_1,2_^+^, are found to be inert towards water; in contrast, the clusters V_*n*≥3_^+^ (especially V_5_^+^ and V_9_^+^) readily react and result in dehydrogenation products, specifically by the reaction ${\mathrm{V}}_{3 \le n \le 30}^ + + {\mathrm{H}}_2{\mathrm{O}} \to {\mathrm{V}}_{n}{\mathrm{O}}^ + + {\mathrm{H}}_2$. Supported by density functional theory (DFT) calculations, we have interpreted the origin of this strongly size-dependent reactivity with its onset at *n* = 3. Analyses of the detailed energetics and reaction dynamics suggest that a three-atom synergistic effect and the presence of wagging vibrations of hydroxyl groups that facilitate the reactions of the formed V–O–V intermediate states and that allow the terminal hydrogen to interact with adsorbed hydrogen atom thus enabling facile hydrogen release (i.e., H_ad_ + H_hydroxyl_ → H_2_). Notably, this mechanism works without having to transfer two hydrogen atoms onto the metal surfaces, similar to an electrochemical Heyrovsky reaction (i.e., ${\mathrm{H}}^ + \left( {{\mathrm{or}}\,{\mathrm{H}}_2{\mathrm{O}}} \right) + {\mathrm{H}}_{{\mathrm{ad}}} + {\mathrm{e}}^ - \to {\mathrm{H}}_2({\mathrm{or}}\,{\mathrm{H}}_2 + {\mathrm{OH}}^ - )$)^20,27,48^. Such three-atom enhanced reactivity was also used to describe the Pt_3_ cluster catalysis in N–H dissociation^49^.

## Results

### Mass spectrometric analysis

**Fig. 1:**
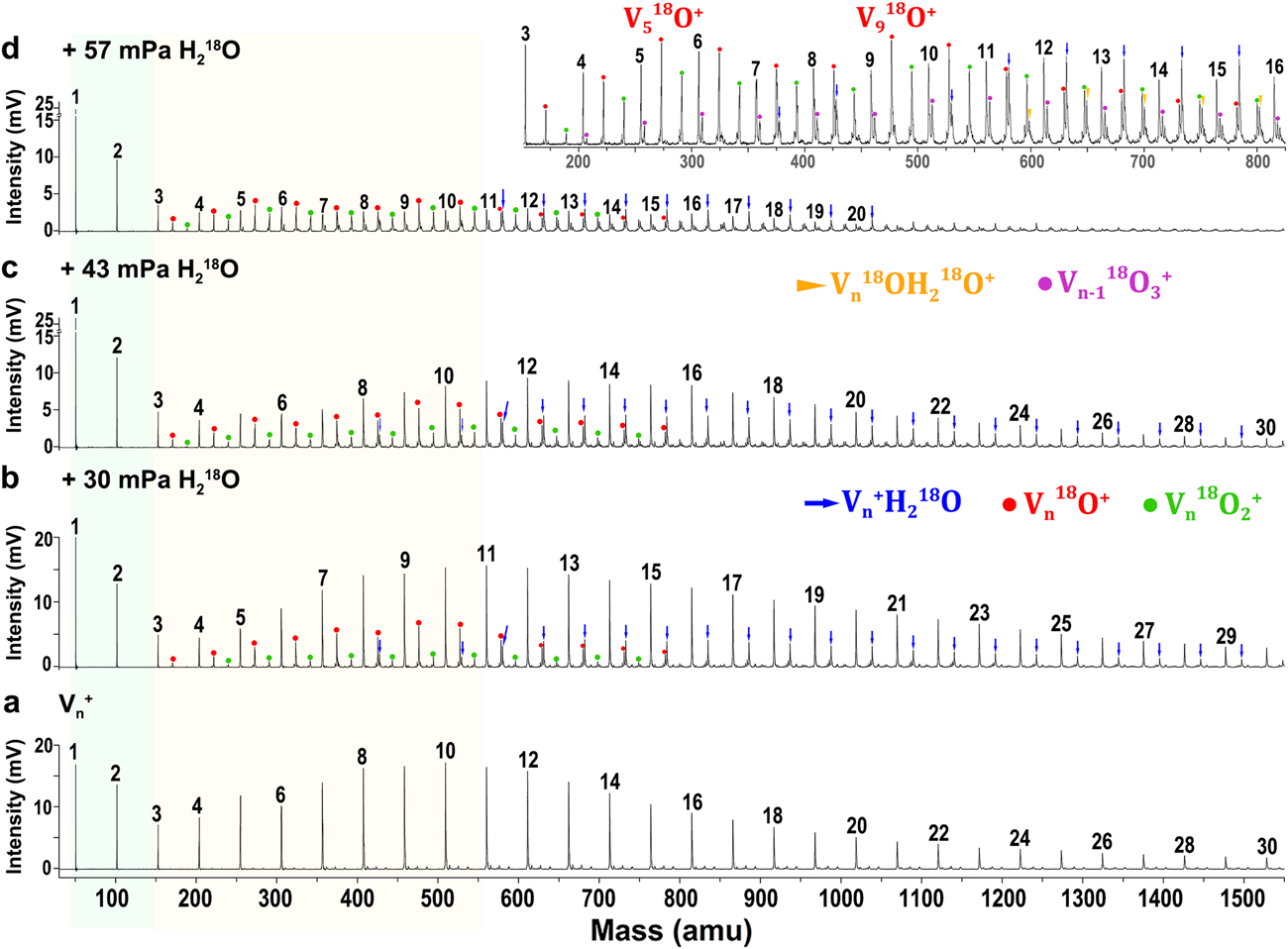
Mass spectrometry observation. Mass spectra of **a** cationic vanadium clusters and **b**–**d** V_*n*_^+^ (*n* = 1–30) reacting with different amounts of H_2_^18^O. The partial pressures of H_2_^18^O vapour are ~30, ~43, and ~57 mPa, respectively, corresponding to an average number of collisions at 4.4, 6.4, and 8.3 × 10^3^ s^−1^. Insets gives a close-up view of **d**. The pure vanadium clusters are labelled by numbers, vanadium monohydrates are labelled by blue arrows, vanadium monoxides are labelled by red circles, vanadium dioxides are labelled by green circles, vanadium trioxides are labelled by purple circles, and hydrated vanadium monoxides are labelled by yellow triangles.

Figure 1a presents a typical mass spectrum of the as-prepared cationic vanadium clusters V_*n*_^+^ (*n* = 1–30) generated in a customized laser evaporation (LaVa) source with He as the buffer gas (1 MPa). To such spectra, different amounts of heavy-oxygen water (H_2_^18^O) were introduced as the reactant. The water vapour (~2% H_2_^18^O in He) was introduced into the (downstream) flow-tube reactor by a pulse valve along with helium carrier gas (0.1 MPa). The reaction products with the thermalized V_*n*_^+^ clusters are shown in Fig. 1b–d. Firstly, the products V_*n*_H_2_^18^O^+^, V_*n*_^18^O^+^, and V_*n*_^18^O_2_^+^ suggest that the adsorption of water and the release of hydrogen occur on the vanadium clusters sequentially. Secondly, we observe that in parallel with an increasing concentration of H_2_^18^O, pure metal clusters are consumed and at the same time, the adsorption and dehydrogenation products emerge gradually. It is clear from this trend that V_*n*_^+^ clusters readily react with water to form vanadium oxides via an adsorption–dissociation process followed by H_2_ release. Thirdly, the spectra can be divided into three regions according to the nature of the products as follows: (i) no reaction product of V_1_^+^ and V_2_^+^ was observed under the conditions of our experiment; (ii) vanadium oxides of V_3–10_^+^ are easily formed with a small number of adsorption products observed; (iii) for V_*n*≥10_^+^, the V_*n*_H_2_^18^O^+^ series dominates the reaction products. We describe these three distinct regions to the size dependence of the reactivity of V_*n*_^+^ clusters with water. (For comparison experiments of the deuterium water and He collision see Supplementary Figs. 3 and 4.)

The inset graph in Fig. 1 gives a close-up view of V_*n*_^+^ (*n* = 3–16) reacting with a relatively large amount of H_2_^18^O in which it can be clearly seen that V_*n*_^+^ clusters react with H_2_O to form V_*n*_H_2_O^+^, V_*n*_O^+^, V_*n*_OH_2_O^+^, V_*n*_O_2_^+^ and V_*n*_O_3_^+^. Among these five types of products, V_*n*_O^+^ and V_*n*_O_2_^+^ shows the highest and second-highest mass abundances, respectively, indicating that H_2_ release dominates the reaction pathways of V_*n*≥3_^+^ with water. In comparison, the relatively lower mass intensities of V_*n*_OH_2_O^+^ presumably correspond to the intermediates of a successive reaction of V_*n*_O^+^ with a second H_2_O molecule that ultimately may lead to the removal of the second H_2_. The observation of V_*n*_O_3_^+^ shows the ability of V_*n*_^+^ to produce three H_2_ molecules by consuming three H_2_O molecules. There are no higher order oxides observed with our experimental condition. The observed reactions can be summarized as

1 $${\mathrm{V}}_n^ + + {\mathrm{H}}_2{\mathrm{O}} \to {\mathrm{V}}_n{\mathrm{H}}_2{\mathrm{O}}^ +,$$

2 $${\mathrm{V}}_n^ + + {\mathrm{H}}_2{\mathrm{O}} \to {\mathrm{V}}_n{\mathrm{O}}^ + + {\mathrm{H}}_2,$$

3 $${\mathrm{V}}_n^ + + 2{\mathrm{H}}_2{\mathrm{O}} \to {\mathrm{V}}_n{\mathrm{OH}}_2{\mathrm{O}}^ + + {\mathrm{H}}_2,$$

4 $${\mathrm{V}}_n^ + + 2{\mathrm{H}}_2{\mathrm{O}} \to {\mathrm{V}}_n{\mathrm{O}}_2^ + + 2{\mathrm{H}}_2,$$

5 $${\mathrm{V}}_n^ + + 3{\mathrm{H}}_2{\mathrm{O}} \to {\mathrm{V}}_n{\mathrm{O}}_3^ + + 3{\mathrm{H}}_2.$$

Considering the above five reaction channels together and noting that the water concentration exceeds the cluster concentration in the beam, the observed reaction of “${\mathrm{V}}_n^ + + m\left( {{\mathrm{H}}_2{\mathrm{O}}} \right) \to {\mathrm{V}}_n{\mathrm{O}}_{x}\left( {{\mathrm{H}}_2{\mathrm{O}}} \right)_{{m} - {x}}^ + \, + \, x{\mathrm{H}}_2\left( {m \ge 1} \right)$” is tentatively described as a pseudo-first-order reaction concerning the depletion of the bare metal clusters. No claim is made about the order of the subsequent reactions. The pseudo-first-order rate constants (*k*_1_) for this reaction can be estimated by the following Eq. (6):

6 $$\ln \frac{{I_{\mathrm{{n}}}}}{{I_{\mathrm{{s}}}}} 	= - k_1\frac{{P_{\mathrm{{e}}}{\Delta} t}}{{k_{\mathrm{{B}}}T}} \\ 	= \ln \frac{{I({\mathrm{V}}_n^ + )}}{{I({\mathrm{V}}_n^ + + {\mathrm{V}}_n{\mathrm{O}}^ + + {\mathrm{V}}_n{\mathrm{H}}_2{\mathrm{O}}^ + + {\mathrm{V}}_n{\mathrm{OH}}_2{\mathrm{O}}^ + + {\mathrm{V}}_n{\mathrm{O}}_2^ + + {\mathrm{V}}_n{\mathrm{O}}_3^ + )}},$$

7 $$k_1^{{\mathrm{{rel}}}}(n) = k_1({\mathrm{V}}_n^ + ){\mathrm{/}}k_1({\mathrm{V}}_{14}^ + )$$

in which the variable *I*_n_ stands for the integrated intensity of the parent peaks at a certain gas pressure, and *I*_s_ refers to the sum of integrated peaks intensities of both the reactants and products. The chemical symbols on the right-hand side represent the measured mass spectrometric intensities. The constants *P*_e_, *k*_B_, and *T* refer to the pressure of the reactant, Boltzmann’s constant, and the reaction temperature (298 K). Δ*t* is the effective residence time in the reactor (∼60 μs). The relative values of the rate constants $k_1^{{\mathrm{{rel}}}}(n)$ defined by Eqs. (6) and (7) are plotted in Fig. 2 (for more details see Supplementary Figs. 1 and 2 and Supplementary Table 1). As is seen, except for V_1_^+^ and V_2_^+^ that have a reaction rate close to zero based on the mass spectrometry observation in this study, the $k_1^{{\mathrm{{rel}}}}$ values of the V_*n*_^+^ (3 ≤ *n* ≤ 20) illustrate size-dependent reaction rates with local smaller values at *n* = 3, 6 and 16. Further, considering the hydrogen evolution channels (Eqs. (2)–(5)) together, also plotted (red) in Fig. 2 is the relative mass abundances of a sum of the HER products (and also seen directly from the mass abundances in Fig. 1), the water dehydrogenation channel has a local maximum for V_5_^+^ and V_9_^+^.

**Fig. 2:**
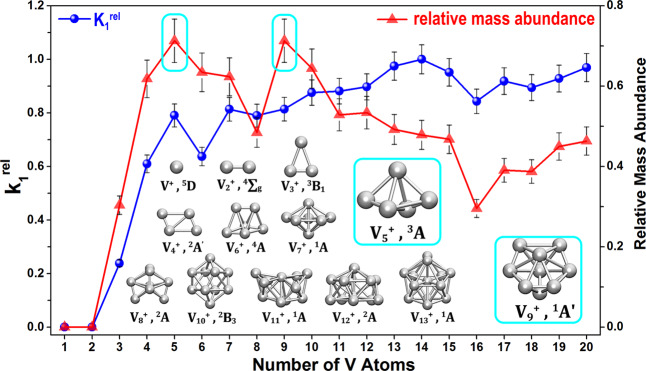
Experimental analysis. (Blue circles) The normalized rate constants relative to the maximum value for the reactions of V_*n*_^+^ (*n* = 1–20) with H_2_^18^O which are defined as $k_1^{{\mathrm{{rel}}}} = k_1({\mathrm{V}}_n^ + ){\mathrm{/}}k_1({\mathrm{V}}_{14}^ + )$. (Red triangles) The relative mass abundances which are defined as $I({\mathrm{V}}_n{\mathrm{O}}^ + + {\mathrm{V}}_n{\mathrm{OH}}_2{\mathrm{O}}^ + + {\mathrm{V}}_n{\mathrm{O}}_2^ + + {\mathrm{V}}_n{\mathrm{O}}_3^ + )$/$I({\mathrm{V}}_n^ + + {\mathrm{V}}_n{\mathrm{O}}^ + + {\mathrm{V}}_n{\mathrm{H}}_2{\mathrm{O}}^ + + {\mathrm{V}}_n{\mathrm{OH}}_2{\mathrm{O}}^ + + {\mathrm{V}}_n{\mathrm{O}}_2^ + + {\mathrm{V}}_n{\mathrm{O}}_3^ + )$. The insets show the structures and electronic states of ground state V_*n*_^+^ clusters. The uncertainties of rate constant estimation and relative intensities are given by the uncertainties in the integration of the mass peaks.

### Structure identification and energetics analysis

**Fig. 3:**
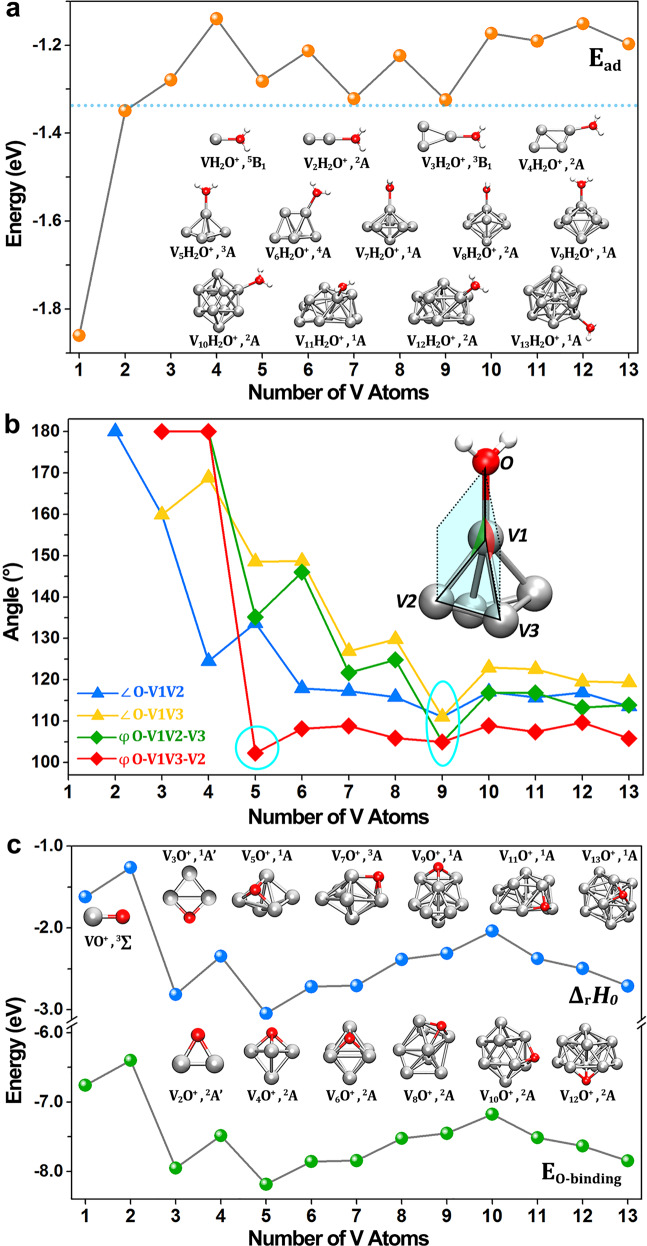
Structures and energetics. **a** Adsorption energy of V_*n*_^+^ and H_2_O ($E_{{\mathrm{{ad}}}} = E\left( {{\mathrm{V}}_n{\mathrm{H}}_2{\mathrm{O}}^ + } \right) - E\left( {{\mathrm{V}}_n^ + } \right) - E\left( {{\mathrm{H}}_2{\mathrm{O}}^ + } \right)$). **b** Bond angles on the lowest energy structures of V_*n*_H_2_O^+^ clusters. The inset gives the definition of bond angle (∠O–V–V, blue and orange dots) and dihedral angle (*φ* O–VV–V, green and red dots). The V atom adsorbing the water molecule is defined as V-1, the V-2 corresponds to the one with the largest positive partial charge, while V-3 is at the minima. **c** The O-binding energies (*E*_O–binding_, green dots) of O to V_*n*_^+^, and the zero-point-vibration corrected total energies of the hydrogen evolution reaction (Δ_*r*_*H*_0_, blue dots) of V_*n*_^+^ (*n* = 1–13) with H_2_O, where the values are just shifted with the same energy determined by the H_2_O reactant and H_2_ product. The insets show the lowest energy structures of V_*n*_H_2_O^+^ and V_*n*_O^+^.

In order to elucidate the origin of the pattern of relative stability, reactivity and the H_2_ release mechanism, ground state structures of V_*n*_^+^, V_*n*_O^+^, and V_*n*_H_2_O^+^ (*n* = 1–13) have been optimized and calculated quantum chemically at the BP86-D3/def2-TZVP level of theory via Gaussian 09 suite of programmes (more results are given in Supplementary Tables 2–6 and Supplementary Figs. 5–7). The lowest energy structures determined for V_*n*_H_2_O^+^ have the following features: (i) water adsorption on V_*n*_^+^ proceeds through vertex V−O coordination modes (Fig. 3a); (ii) the electronic and geometric structures of the V_*n*_^+^ clusters are preserved to a large extent when forming V_*n*_H_2_O^+^. In addition, the adsorption energies of H_2_O onto V_*n*_^+^ ($E_{{\mathrm{{ad}}}} = E\left( {{\mathrm{V}}_n{\mathrm{H}}_2{\mathrm{O}}^ + } \right) - E\left( {{\mathrm{V}}_n^ + } \right) - E\left( {{\mathrm{H}}_2{\mathrm{O}}^ + } \right)$) display a dramatic size dependence. The *E*_ad_ of V_1_^+^ is substantially higher than the values for the V_*n*_^+^ clusters, indicating the relative stability of V_1_H_2_O^+^ and hence explains the suppression of H_2_ release from the complex. This V_*n*_H_2_O^+^ binding energetics is in concordance with a previous study^46^, the water-binding energies decrease from *n* = 1 to 4 and show an odd–even oscillation for *n* = 3–13. Considering that the positions of the water molecules are quite external from the clusters, without any significant rearrangement of the cluster geometry, the odd–even effect in the binding energy of the water molecule to the V_*n*_^+^ cluster reflects a similar effect in the bare cluster dissociation energy, albeit with a factor-of-ten smaller amplitude. Similar odd–even effects have been seen in clusters of alkali and coinage metals^50–52^, where the prevailing interpretation is that it arises as a consequence of Jahn–Teller distortion combined with oscillating spin degeneracy.

It is noteworthy that the most exothermic reaction appears at the size (*n* = 5) where also 3D structures appear as the energy minima found in this study. The close relation between geometry and reactivity surmised is corroborated by Fig. 3b which summarizes the bond angle of O–V–V and the dihedral angle of O–VV–V in the V_*n*_H_2_O^+^ series. Generally speaking, the smaller the ∠O–V–V and φ O–VV–V values are, the more easily the H_2_O molecule will bend over a triangular face of V_*n*_^+^ and hence promote HER processes. Conversely, the linear shape of V_1_H_2_O^+^ and V_2_H_2_O^+^ and the strong interaction between metal and water impede the bending of the V–O bond. In comparison, the dihedral angle *φ* O–VV–V in V_5_H_2_O^+^ is the smallest among the V_*n*_H_2_O^+^ series, indicating an efficient HER process leading to the formation of V_5_O^+^. Also, V_9_H_2_O^+^ has the smallest bond angle ∠O–V–V in the series, as well as a relatively small dihedral angle *φ* O–VV–V, which facilitates wagging vibrations of the V–OH_2_ (Supplementary Fig. 8) and thus benefits the formation of V–O–V intermediate states according to this scheme. This provides a reasonable explanation for the preponderance of V_9_O^+^ products in the mass spectra.

Figure 3c presents the lowest energy structures of the V_*n*_O^+^ clusters after the hydrogen release, as well as the O-binding energies ($E_{\mathrm{{{O - binding}}}} = E\left( {{\mathrm{V}}_n{\mathrm{O}}^ + } \right) - E\left( {{\mathrm{V}}_n^ + } \right) - E\left( {\mathrm{O}} \right)$) and thermodynamic energy changes for the hydrogen evolution reaction (Δ_*r*_*H*_0_) defined by ${\Delta} _rH_0 = [E\left( {{\mathrm{V}}_n{\mathrm{O}}^ + } \right) + E\left( {{\mathrm{H}}_2} \right)] - [E\left( {{\mathrm{V}}_n^ + } \right) + E\left( {{\mathrm{H}}_2{\mathrm{O}}} \right)]$, where the *E* all correspond to the zero-point-vibration corrected total energies in their ground states. This figure therefore shows the thermodynamics tendency of the H_2_ evolution with size. It is seen that V–O shows a bonding energy at about 6.76 eV, which is consistent with the previously published studies of dissociation energy of V–O bond at ~6.48 ± 0.09 eV^53,54^, although the value deduced from threshold analysis of dissociation reaction at 5.99 eV^54^, due to likely systematic error on nonthermalized state. V_2_O^+^ and V_3_O^+^ take on a planar V–O–V structure with *C*_2*v*_ symmetry, which can be constructed by capping an oxygen atom on the V–V edge. In contrast, for all the other V_*n*_O^+^ (*n* = 4–13) clusters, the O atom is attached to one of the triangular faces of V_*n*_^+^. Furthermore, the energy release for reaction with V_1_^+^ and V_2_^+^ is much less than that of V_3_^+^ to V_13_^+^, which supports the absence of VO^+^ and V_2_O^+^ in the mass spectra. DFT-calculated energetics including V-atom dissociation energies, H_2_O-binding energy and O-binding energies etc., along with a comparison to those in literatures, are provided in Supplementary Tables 7–10 and Supplementary Figs. 9–12. Besides, a reasonable HER of water on metals is associated with the ability to donate (or accept) electrons to (or from) the molecules, including initial steps of the “${\mathrm{V}}_n^ + + {\mathrm{H}}_2{\mathrm{O}}$”, the activation and rupture of the O–H bond. From the natural population analysis (NPA) of charges on H_2_O in V_*n*_H_2_O^+^ clusters (Supplementary Fig. 13), it is seen that V_9_^+^ has the highest amount of electron transfer, consistent with its outstanding reactivity. More details showing the energy decomposition analysis is provided in Supplementary Table 12 and Supplementary Fig. 16.

### HER mechanism

**Fig. 4:**
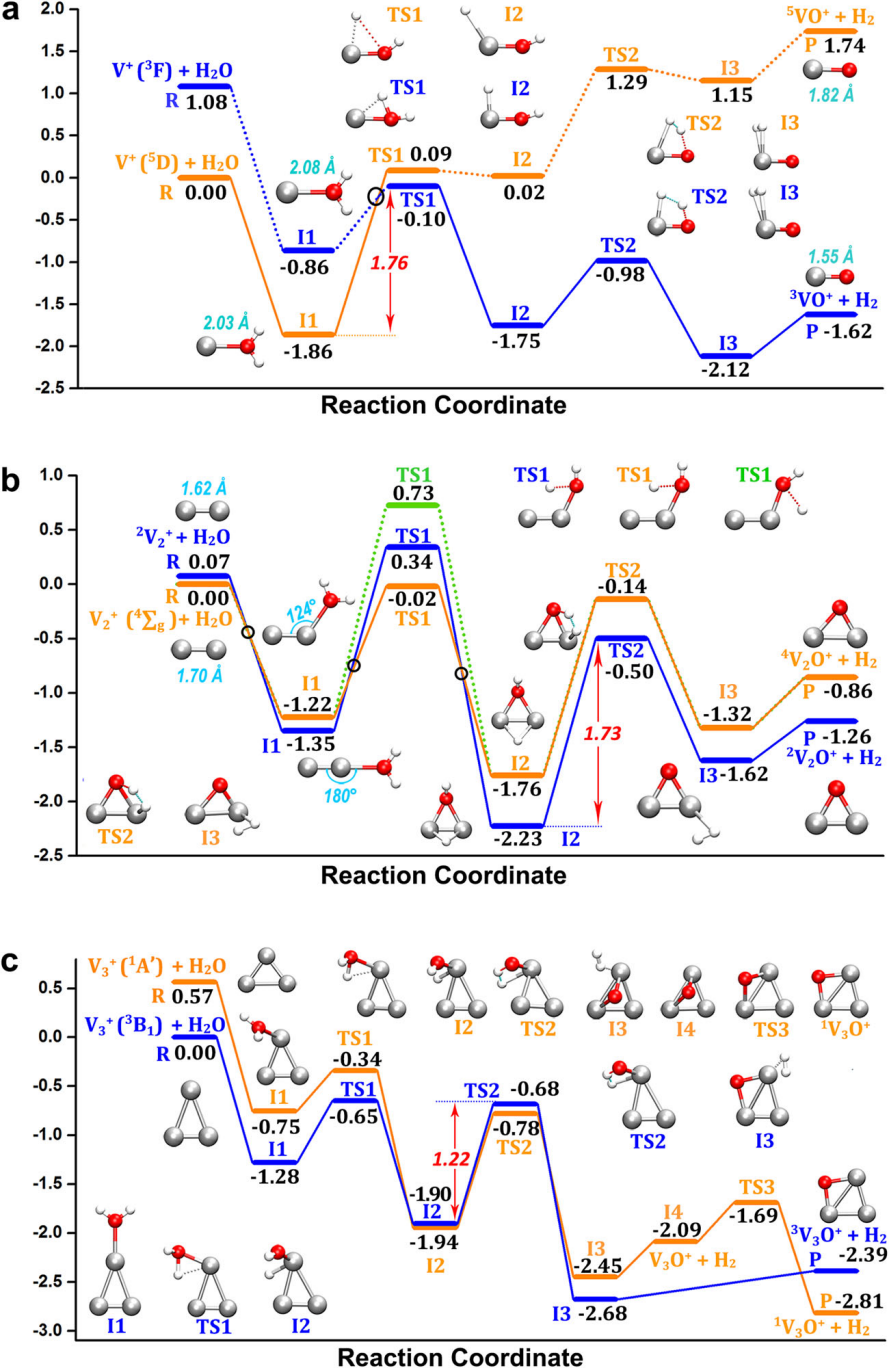
Reaction coordinates of HER on V_1–3_^+^. Reaction coordinates of **a** “${\mathrm{V}}_1^ + + {\mathrm{H}}_2{\mathrm{O}} \to {\mathrm{V}}_1{\mathrm{O}}^ + + {\mathrm{H}}_2$”, **b** “${\mathrm{V}}_2^ + + {\mathrm{H}}_2{\mathrm{O}} \to {\mathrm{V}}_2{\mathrm{O}}^ + + {\mathrm{H}}_2$” and **c** “${\mathrm{V}}_3^ + + {\mathrm{H}}_2{\mathrm{O}} \to {\mathrm{V}}_3{\mathrm{O}}^ + + {\mathrm{H}}_2$”. The bond lengths and bond angles are labelled in cyan. The energy values are relative to the entrance channel, corrected with zero-point vibration energies, and given in eV. Spin multiplicity is marked as pre-superscript. The black circles stand for spin inversion.

Having determined the thermodynamics of HER of these V_*n*_^+^ clusters, Fig. 4 presents a comparison of the energetics of the reaction coordinates for “${\mathrm{V}}_{1 - 3}^ + + {\mathrm{H}}_2{\mathrm{O}} \to {\mathrm{V}}_n{\mathrm{O}}^ + + {\mathrm{H}}_2$”. For V^+^ (Fig. 4a), the ground electronic state is V^+^(^5^D) which displays a M^+^–OH_2_ bond energy of 1.86 eV, which is consistent with the previous studies by Armentrout and colleagues^55^. The adsorption products (I_1_) comply with the spin conservation for triplet and quintet potential energy surfaces (PESs), while the TS_1_ of spin-triplet state lies below the energy of the spin-quintet state by 0.19 eV, likely leading to a spin crossing before the subsequent reaction steps. However, there remains the large energy barrier of 1.76 eV for the hydrogen atom transfer to V^+^ required for the production of the HV^+^OH intermediate (I_2_). The second hydrogen transfer from oxygen to the metal takes place through TS_2_. This transition structure leads to another intermediate found on the reaction path, the H_2_VO^+^ complex (I_3_). From this intermediate, the loss of H_2_ proceeds without a transition structure to the observed major products, VO^+^ and H_2_^40^. It should be noted that there is a V^+^(^3^F) state which is 1.08 eV higher in energy than V^+^(^5^D), consistent with previously reported results^44,45^. Nevertheless, an initial spin excitation of V^+^(^5^D) to V^+^(^3^F) could promote the HER processes, as was observed in the previous study involving black body radiation^41^; otherwise, the thermalized downstream reaction of ground-state V^+^(^5^D) with water could not be enabled even though this reaction is exothermic. Considering that the clusters in this study are close to thermal equilibrium, and that under the ambient conditions, the 1.76 eV energy barrier is unsurmountable for thermalized V^+^ ions due to the good equilibration with the helium buffer gas, a total reaction exothermicity notwithstanding. The large energy barrier of the transition structure for the first-step hydrogen transfer was also identified by previous studies, e.g., with a value of 2.23 eV in the work of A. Irigoras et al. ^40^. Therefore, the large energy barrier in the first-step hydrogen transfer impedes the production of triplet VO^+^, in good agreement with our mass spectrometry observations.

For V_2_^+^, we investigated two reaction pathways, considering the spin multicity of V_2_^+^, as shown in Fig. 4b where a blue line indicates the path of doublet V_2_^+^ and an orange the quartet V_2_^+^ path. Between I_1_ and TS_1_, the two spin states change energy-ordering resulting in spin crossing for the blue and orange paths. Considering that the energies of quartet and doublet V_2_^+^ are close (0.07 eV), the first hydrogen transfer is a two-state step^56^ with spin inversion occurring twice. In addition, another possible path is offered by transferring the first H atom to the V atom (^4^V_2_^+^, green line). The rate-determine step in the reaction of “${\mathrm{V}}_2^ + + {\mathrm{H}}_2{\mathrm{O}}$” is the second hydrogen transfer with a barrier of 1.73 eV which is difficult to cross, mainly due to the stability of I_2_ intermediate state. Thus, we ascribe the absence of V_2_O^+^ in the mass spectra to the large energy barriers of transition structures although overall this reaction itself is thermodynamically favourable. We reiterate the observation that the presence of the helium gas plays an essential role for this conclusion. In the absence of a thermalizing agent, the determining parameter would be the overall exothermicity of the reaction.

The case of V_3_^+^ (Fig. 4c) represents a very different situation from that of V_1,2_^+^. The spin multiplicity for the lowest energy structures of ^1^V_3_O^+^, also differs from that of the bare cluster, ^3^V_3_^+^, and two reaction pathways are considered. A difference to V_1,2_^+^ is that the first hydrogen barrier is much lower for V_3_^+^ than that of V_1,2_^+^ due to a significantly decreased bond angle ∠O–V–V in V_3_H_2_O^+^, as discussed above. This allows wagging vibrations of the hydroxyl group and thus the formation of V–O–V intermediates. The second hydrogen transfer is the rate-determine step with a barrier of 1.22 eV. Also, this value is smaller than the rate-determine step in V_1,2_^+^, indicating a feasible HER for V_3_^+^ clusters. For reaction coordinate of “${\mathrm{V}}_3^ + + 2{\mathrm{H}}_2{\mathrm{O}} \to {\mathrm{V}}_3{\mathrm{O}}_2{\mathrm{H}}_2^ + + {\mathrm{H}}_2$” see Supplementary Fig. 17.

**Fig. 5:**
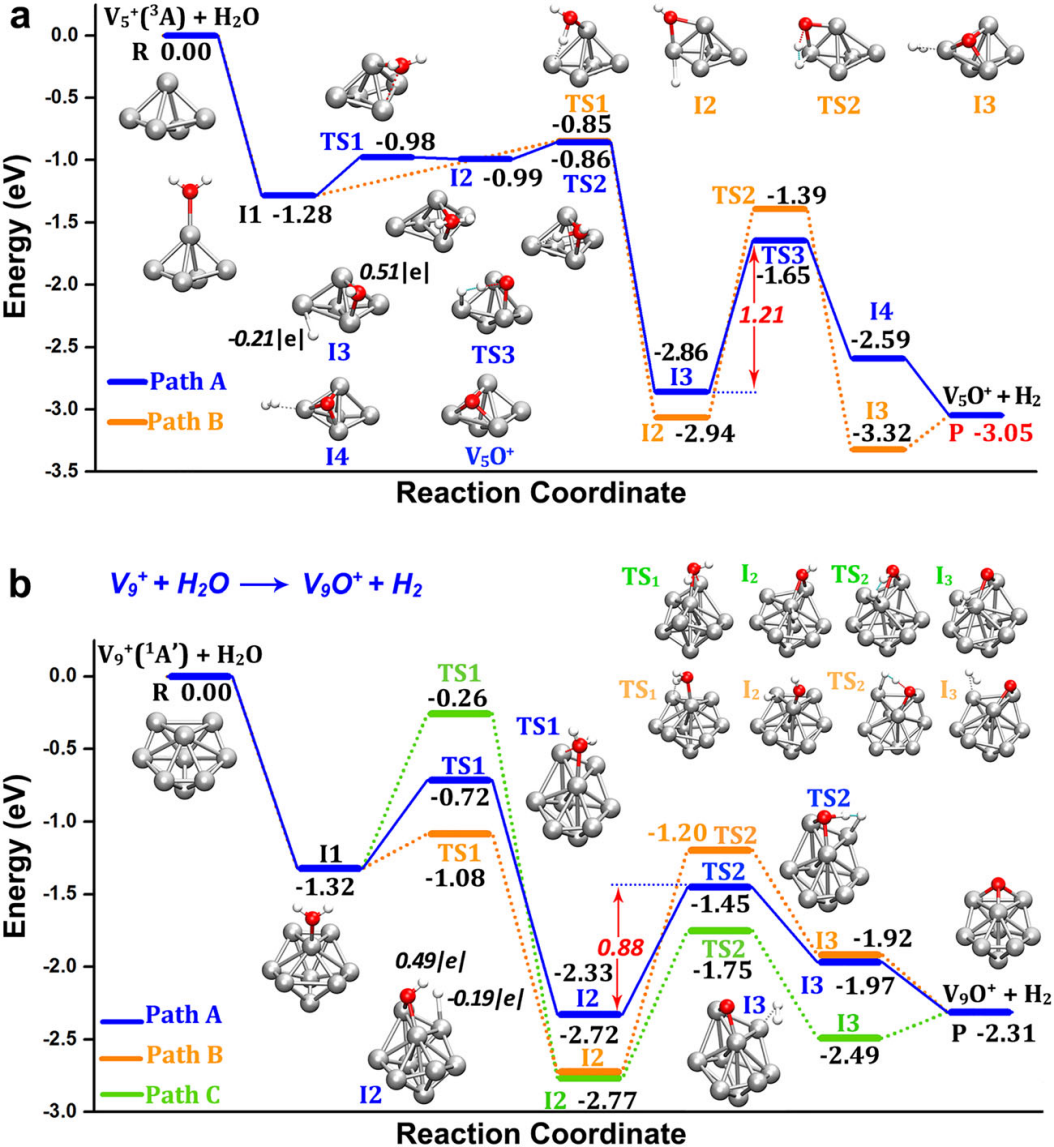
Reaction coordinates of HER on V_5_^+^ and V_9_^+^. **a** The reactions of “${\mathrm{V}}_5^ + + {\mathrm{H}}_2{\mathrm{O}} \to {\mathrm{V}}_5{\mathrm{O}}^ + + {\mathrm{H}}_2$”, and **b** “${\mathrm{V}}_9^ + + {\mathrm{H}}_2{\mathrm{O}} \to {\mathrm{V}}_9{\mathrm{O}}^ + + {\mathrm{H}}_2$”. The energy values are relative to the entrance channel and are given in eV. The marked partial charges on hydrogen atoms of the intermediates (HV_5_OH^+^ and HV_9_OH^+^) are calculated at BP86-D3/def2-TZVP level of theory using NBO 6.0 method^73^.

The reaction of V_5_^+^ shows a similar behaviour. Figure 5a displays the reaction coordinates for the reaction ${\mathrm{V}}_5^ + + {\mathrm{H}}_2{\mathrm{O}} \to {\mathrm{V}}_5{\mathrm{O}}^ + + {\mathrm{H}}_2$, where the small dihedral angle of O–VV–V in the ground state structure of V_5_H_2_O^+^ benefits the first hydrogen transfer to produce the HV_5_OH^+^ intermediate (I_3_, blue line). In this intermediate state, the O atom bridges two V atoms and the H atom is connected with the third V atom in the V–V–V plane. Subsequently H_2_ releases from the third V atom via TS_3_ with an energy barrier of 1.21 eV. The relatively low transition structure energy and the largely exothermic final product V_5_O^+^ accounts for its reasonable mass abundance in the experimental observation. It is important to notice that the reaction pathway without the participation of the third V atom in the triangle (orange line) has a larger barrier in the rate-determining step, which once more illustrates the importance of cooperation of the third vanadium atom in the V_*n*_^+^ cluster reactions with water. Note that the LUMO energy level of V_5_^+^ is close to HOMO level of H_2_O, which could benefit the V_5_^+^–H_2_O orbital interactions (Supplementary Figs. 14 and 15).

To investigate the generality of this principle, we have also calculated the reaction coordinates for “${\mathrm{V}}_9^ + + {\mathrm{H}}_2{\mathrm{O}} \to {\mathrm{V}}_9{\mathrm{O}}^ + + {\mathrm{H}}_2$”, which is shown in Fig. 5b. Among the three possible pathways, the one marked with blue line exhibits a fairly low barrier (0.88 eV) from HV_9_OH^+^ (I_2_) to H_2_V_9_O^+^ (I_3_). Again, the pathway of V_9_^+^ HER demonstrates the importance of the presence of three V atoms, two of which form a V–O–V bridge in the H_2_V_*n*_O^+^ intermediate and the third provides the hydrogen receptor and the H_2_ removal site. Similar to the reaction paths of “${\mathrm{V}}_5^ + + {\mathrm{H}}_2{\mathrm{O}}$”, the O-atom capping on the V–V–V triangle plane is formed in H_2_V_9_O^+^ after a two-step hydrogen transfer with the second step the rate-determining one. The mass spectrometry observation (Fig. 1) reveals that V_9_O^+^ dominates the reaction products, with rare V_9_(H_2_O)^+^ product being observed, which coincides with the fairly low barrier and also large NPA charge transfer in V_9_(H_2_O)^+^ (Supplementary Fig. 13 and Supplementary Table 11), indicating high HER activity of V_9_^+^. In comparison, there is relatively lower HER activity for V_*n*_^+^ with *n* > 10, although they do react and form water-addition products. This could be associated with the higher density of vibrational states, providing a more efficient dissipation of the association energy, in combination with a longer time for H atoms to reach the proper site to complete H–H recombination.

## Discussion

The gas-phase reactivity of cationic vanadium clusters V_*n*_^+^ towards water has been studied experimentally and theoretically. A significant H_2_ release is observed for *n* ≥ 3, deduced from reaction products observed in the mass spectra. Analyses of the mass abundances, energetics and reaction dynamics indicate the inertness of V_1,2_^+^ towards water at room temperature. In contrast, the HER is significant for V_3_^+^, V_5_^+^ and V_9_^+^, processes that point to the H_2_ release mechanism from such transition metal cluster cations. The first step in the HER is the formation of the V_*n*_H_2_O^+^ complex, followed by the displacement of one hydrogen atom from oxygen to the metal cluster, reaching the HV_*n*_^+^OH molecule through a transition structure TS_1_. This dissociative chemisorption step has similarities to the reaction of main group metal clusters with alcohols, but still differs as the transition metal clusters arrange with a V–O–V bridge for the I_2_ intermediate state. At the second transition structure, the remaining hydrogen atom can either transfer from oxygen to the metal through a second transition structure TS_2_ or interact directly with the adsorbed H atom. Both cases lead to a facile HER process “H_ad_ + H_hydroxyl_ → H_2_”. Furthermore, the final intermediates of H_2_V_*n*_O^+^ complexes allow the oxygen to bond with three vanadium atoms, which is associated with a highly exothermic product. In this mechanism, the bond angle of O–V–V and dihedral angle of O–VV–V in V_*n*_H_2_O^+^ complex plays a pivotal role for the HER reaction process. The formation of a V–O–V bridge and the participation of the third V atom for H attachment in HV_*n*_^+^OH molecule are the determinants of the energy barriers. The distinct difference from the reactions of aluminium-based clusters is that a V_*n*_^+^ cluster only needs one water molecule to generate a hydrogen molecule. Thus, this study not only updates the HER mechanism on metals, but also hints at a strategy to design materials that could supply portable fuel cells of hydrogen.

## Methods

### Experimental methods

The home-made reflection time-of-flight mass spectrometer (Re-TOFMS)^47^ equipped with a fast-flow reaction tube was utilized to conduct the gas-phase experiments of the cationic vanadium clusters V_*n*_^+^ reacting with heavy-oxygen water (2% H_2_^18^O/He). The heavy oxygen isotope (oxygen-18) was used to clear the mass spectroscopic identification of water reaction products without interference of trace oxygen contamination (oxygen-16) in the vacuum chamber. A brief description of the apparatus is given here while detailed information can be found in our previously published studies^44,57^. The vanadium clusters (V_*n*_^+^) were generated in the cluster formation channel after laser ablation of a vanadium disk (99.9% purity), with He (99.999%, 1.0 MPa backing pressure) as the buffer gas. After ablation and cluster generation, the molecular beam flowed through a nozzle to the reaction tube where 2% H_2_^18^O/He was injected by a pulsed general valve (Parker, Serial 9). The amount of H_2_^18^O was controlled by varying the on-time pulse width of the reaction gas injection. At the end of the reaction chamber, the molecular beam was skimmed, differentially pumped and entered the TOF chamber for mass spectrometry analysis. The molecular density of the reactant gas (*ρ*, molecule m^−3^) was experimentally controlled and determined by *ρ* = *N*/(*t·ʋ·S*), in which *N* is the number of reaction gas molecule per pulse, *t* is the pulse duration, *S* and *ʋ* are the cross-sectional areas defined by the inner diameters of the reaction-tube and the flowing velocity of cluster beam in the reactor. From these numbers, the value of *ρ* in this study were calculated to be $0.7\sim 1.4 \times 10^{19}\,{\mathrm{molecule}}\,{\mathrm{m}}^{ - 3}$, and the corresponding partial pressure (*P*) of reaction gas was 30–57 mPa for the different valve-opening times.

### Theoretical methods

Theoretical calculations were conducted using the generalized gradient approximation (GGA) BP86 functional^58,59^ with the DFT-D3 dispersion correction^60^ included. All the structure optimization and reaction coordination research were performed using the def2-TZVP basis sets^61,62^ implemented in the Gaussian 09 programme^63^. The BP86 functional has been proved to provide accurate geometries and spin states for transition metals including vanadium^44,64,65^. We investigated a large number of possible structures, including the structures previously found for vanadium clusters^66–68^, vanadium hydrates^40,46,69^, and vanadium oxides^70,71^. Multiple spin configurations and vibrational frequency calculations were examined to ensure that the lowest energy structures and multiplicity were correctly identified. The convergence threshold for the RMS forces in the optimization was set to be 10^−4^ a.u., and all the procedures meet this criterion. All energies in this work were corrected for the vibrational zero-point energies. For the determination of transition structures (TSs), we applied both the Berny algorithm and synchronous transit guided quasi-Newton (STQN) methods. The initial approximate transition structures were obtained by relaxed PES scans using an appropriate internal coordinate. For the candidate TSs, intrinsic reaction-coordinate (IRC) calculations were conducted to check the connection of a TS with both-side local minima; also, the number of vibrational imaginary frequencies was examined and the ascertained TSs had only one imaginary frequency. The orbital patterns are plotted via the software package of visual molecular dynamics (VMD)^72^.

## Supplementary information

Supplementary Information

## Data Availability

All data supporting the findings of this study are available within the paper and its supplementary information.
