# Supplementary material for: Hydrogen release from a single water molecule on Vn+ (3 ≤ n ≤ 30)
Source: Commun Chem. 2020 Oct 30;3:148. doi: 10.1038/s42004-020-00396-9 (PMC9814650; doi:10.1038/s42004-020-00396-9)
Supplement: Supplementary file 1 — Supplementary Information [file 42004_2020_396_MOESM1_ESM.pdf]

Supporting Information for

Hydrogen Release from a Single H_2O Molecule on V_n^+ ($3 \leq n \leq 30$)

Hanyu Zhang,[1] Haiming Wu,[1] Yuhan Jia,[1,2] Baoqi Yin,[1,2] Lijun Geng,[1] Zhixun Luo[1,2,*]
and Klavs Hansen[3]

[1] Beijing National Laboratory of Molecular sciences (BNLMS), State Key Laboratory for Structural Chemistry of Unstable and Stable Species, Institute of Chemistry, Chinese Academy of Sciences; Beijing 100190, P. R. China.

[2] University of Chinese Academy of Sciences, Beijing 100049, P. R. China.

[3] Joint Centre for Quantum Studies and Department of Physics, School of Science, Tianjin University, Tianjin, China.

Corresponding author: Zhixun Luo, Email: zxluo@iccas.ac.cn

H. Z. and H. W. contributed equally to this work.

Contents

S1 Experimental Details of "V_n^+ + $H_2{}^{18}O$"....2

S2 Reactions of "V_n^+ + D_2O"....5

S3 Collision Experiments of "V_n^+ + He"....6

S4 Structure Optimization....7

S5 DFT-Calculated Vibration Modes....15

S6 Energetics....16

S7 Natural Population Analysis of Charges....21

S8 Frontier Orbitals....23

S9 Energy Decomposition Analysis....24

S10 Reaction Coordinate of "V_3^+ + $2H_2O$"....27

Supplementary References....27

S1 Experimental Details of "V_n^+ + $H_2^{18}O$"

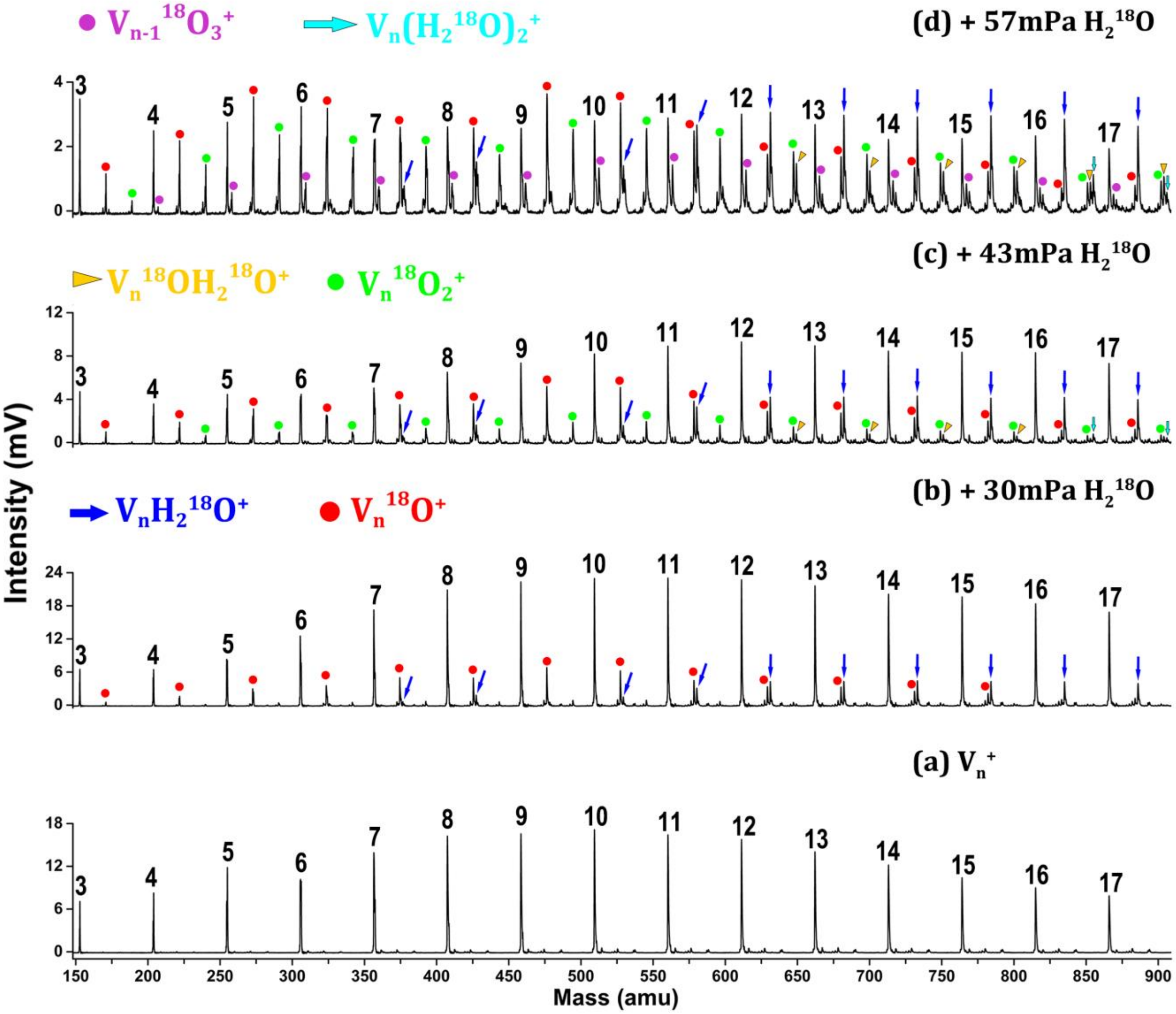

Supplementary Figure 1 Mass spectra of **(a)** cationic vanadium clusters and **(b-d)** V_n^+ reacting with different amount of $H_2{}^{18}O$. The pure vanadium clusters are labelled by numbers, vanadium monohydrates are labelled by blue arrows, vanadium monoxides are labelled by red circles, vanadium dioxides are labelled by green circles, vanadium trioxides are labelled by purple circles, and hydrated vanadium monoxides are labelled by yellow triangles.

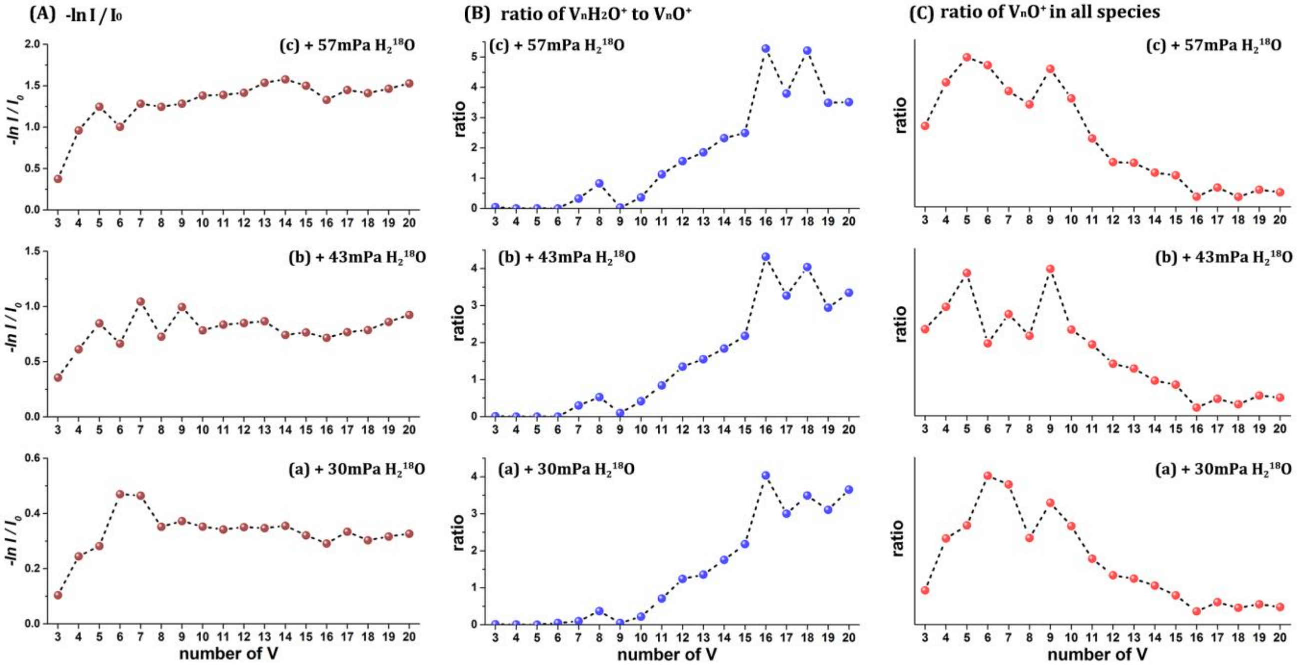

Supplementary Figure 2 Mass abundance analysis for the reactions of V_n^++$H_2{}^{18}O$ with different $H_2{}^{18}O$ doses, based on the equations: **(A)** $-ln\frac{V_n^+}{V_n^+ + V_n{}^{18}O^+ + V_nH_2{}^{18}O^+ + V_n{}^{18}OH_2{}^{18}O^+ + V_n{}^{18}O_2^+ + V_n(H_2{}^{18}O)_2^+ + V_n{}^{18}O_3^+}$, **(B)** $\frac{V_nH_2{}^{18}O^+}{V_n{}^{18}O^+}$, and **(C)** $\frac{V_n{}^{18}O^+}{V_n^+ + V_n{}^{18}O^+ + V_nH_2{}^{18}O^+ + V_n{}^{18}OH_2{}^{18}O^+ + V_n{}^{18}O_2^+ + V_n(H_2{}^{18}O)_2^+ + V_n{}^{18}O_3^+}$.

Supplementary Table 1 The estimated rate constants of V_n^+ clusters in reacting with $H_2{}^{18}O$.

V_n^+ clusters	Rate coefficients ($cm^3 \cdot molecule^{-1} \cdot s^{-1}$)
1	~1.1 $\times 10^{-11}$ (Bohme's work)[1]
	< 10^{-11}
2	<10^{-11}
3	4.50×10^{-10}
4	1.15×10^{-9}
5	1.49×10^{-9}
6	1.20×10^{-9}
7	1.54×10^{-9}
8	1.49×10^{-9}
9	1.54×10^{-9}
10	1.65×10^{-9}
11	1.66×10^{-9}
12	1.69×10^{-9}
13	1.84×10^{-9}
14	1.89×10^{-9}
15	1.80×10^{-9}
16	1.59×10^{-9}
17	1.73×10^{-9}
18	1.69×10^{-9}
19	1.75×10^{-9}
20	1.83×10^{-9}

Note: These rate constants are estimated based on the following parameters: The partial pressure of water in the reaction tube is 57 mPa, the reaction gas ($H_2{}^{18}O$) molecule density in the reaction tube is 1.39×10^{19} molecule$\cdot m^{-3}$, the effective residence time in the reactor is 60 μs. The reaction temperature is 298 K.

S2 Reactions of "$V_n^+ + D_2O$"

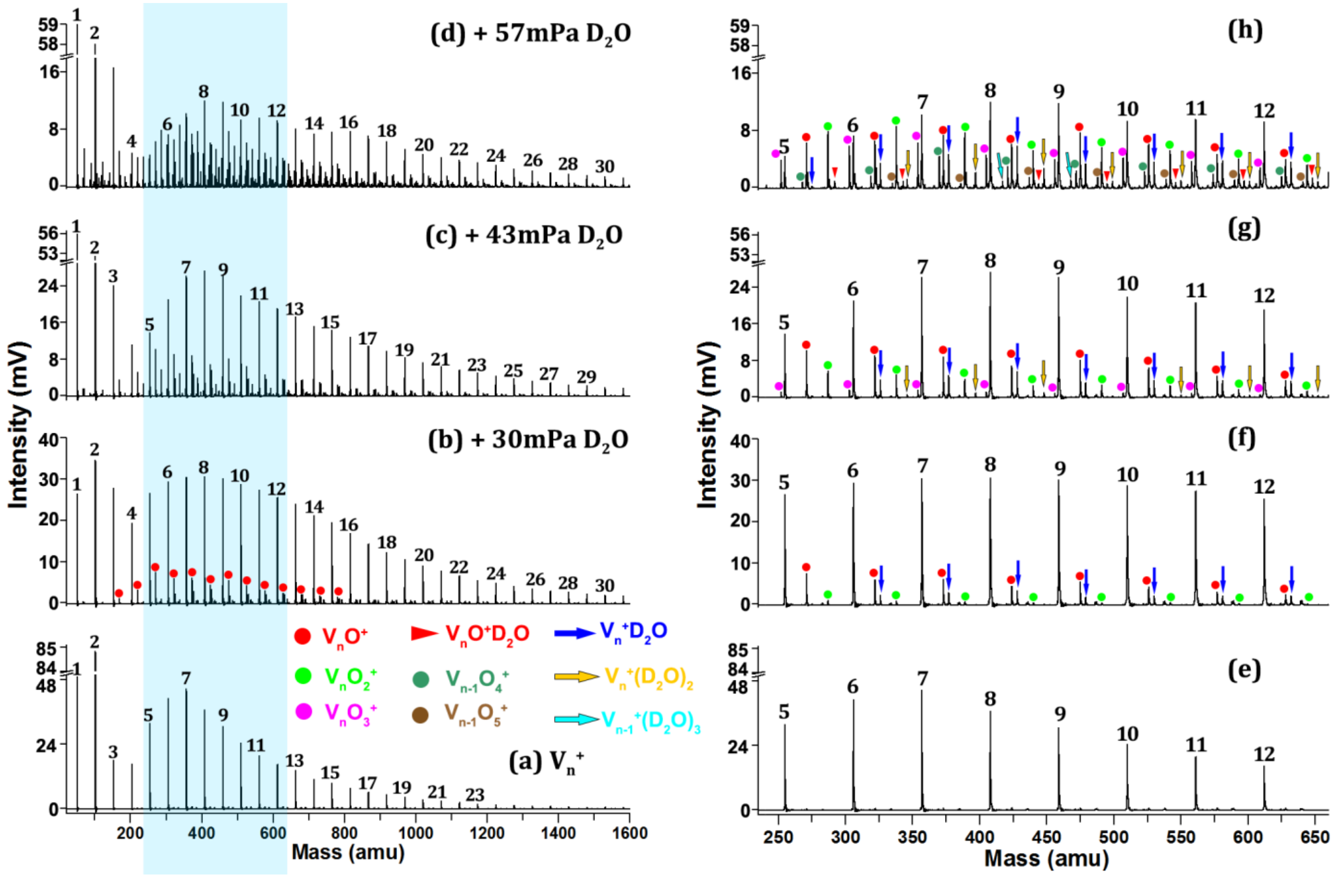

Supplementary Figure 3 Mass spectra of **(a)** cationic vanadium clusters and **(b-d)** V_n^+ reacting with different amount of D_2O, with the expanded areas shown in **(e-h)** respectively. The pure vanadium clusters are labelled by numbers.

S3 Collision Experiments of “V_n^+ + He”

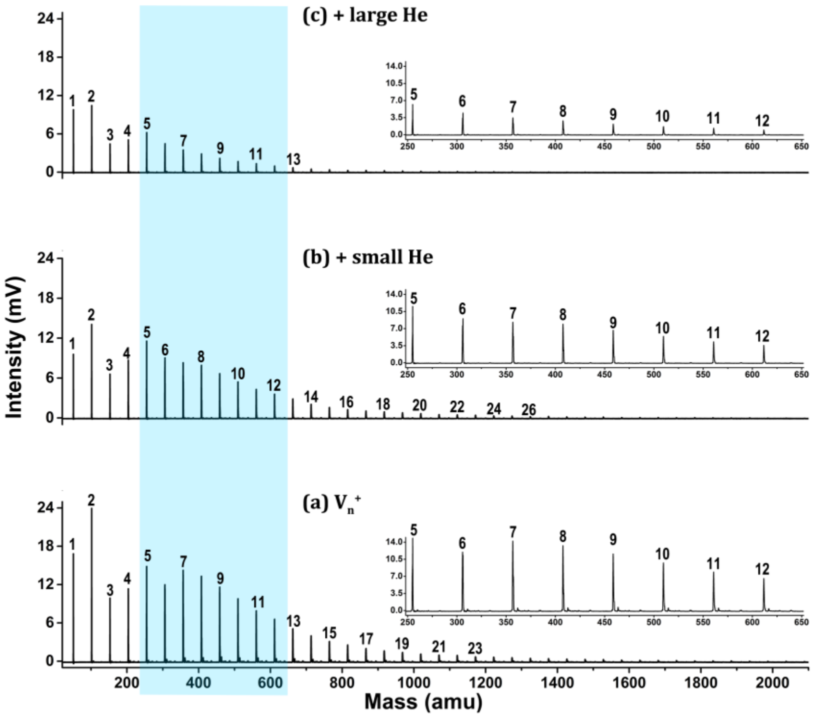

Supplementary Figure 4 Mass spectra of **(a)** the cationic vanadium clusters produced with helium as buffer gas, **(b-c)** and after suffering from the downstream collision experiments by introducing pure helium gas as reactant in place of H_2O/He mixture. The V_n^+ clusters are labelled by numbers.

S4 Structure Optimization

Supplementary Table 2 Relative energies of V_n^+ (n=1-13) isomers, calculated at BP86-D3/def2-TZVP level of theory. Energies are given in in eV. Values of M refer to spin multiplicity.

**V_n^+**		**Energies**			
		M=1	M=3	M=5	M=7
**n=1**		2.73	1.08	**0.00**	33.87
**n=3**		0.57	**0.00**	0.29	2.13
**n=5**	a	0.22	**0.00**	0.20	0.57
	b	0.47	0.52	1.31	
**n=7**	a	**0.00**	0.13	0.43	0.95
	b	0.26	0.11	0.37	0.83
	c	0.71	0.71	0.78	
	d	0.78	0.73	0.78	1.09
**n=9**	a	**0.00**	0.04	0.28	
	b	0.30	0.36	0.63	
**n=11**	a	**0.00**	0.03	0.69	
	b	1.23	0.75	1.18	
**n=13**	a	**0.00**	0.06	0.29	
	b	1.27	1.27	1.28	
	c	0.82	0.78	1.08	

**V_n^+**		**Energies**			
		M=2	M=4	M=6	M=8
n=2		0.07	**0.00**	1.61	1.60
n=4	a	**0.00**	0.34	0.97	2.16
	b	0.70	0.59	0.88	1.23
n=6	a	0.67	**0.00**	0.65	
	b	0.82	0.49	0.76	1.39
n=8	a	**0.00**	0.47	2.02	
	b	0.18	0.60	1.18	
	c	0.50	0.49	0.88	
	d	0.59	0.91	2.42	
	e	1.42	1.64	1.75	
n=10	a	**0.00**	0.59	1.19	
	b	0.89	1.09	1.73	
n=12	a	**0.00**	0.18	0.61	
	b	0.01	0.75	1.54	
	c	0.30	0.48	0.81	
	d	0.47	1.39	1.39	

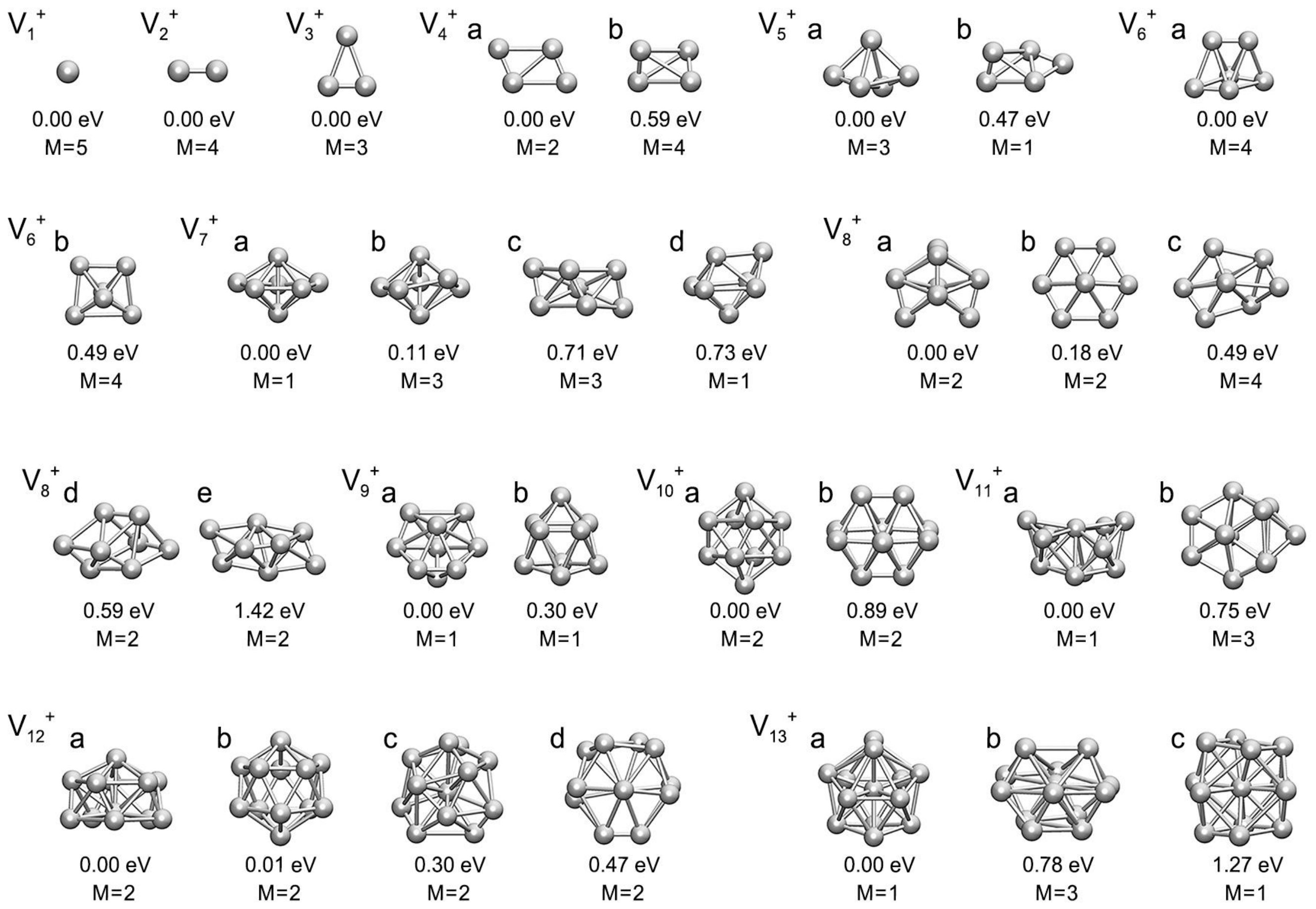

Supplementary Figure 5 Structures of the V_n^+ (n=1-13) isomers in Supplementary Table 2 and their relative energy difference (in eV), calculated at BP86-D3/def-2TZVP level. M indicates spin multiplicity.

Supplementary Table 3 A comparation of the relative energies of vanadium cluster isomers (taking V_5^+ and V_9^+ as example) at different convergence thresholds. The calculations are conducted using Gaussian 09 program at BP86-D3/def2-TZVP level. The energies are corrected with zero-point vibration energies and given eV. Values of M refer to spin multiplicity.

**V_n^+ Cluster**		**Relative Energies (eV)**			
		M=1	M=3	M=5	M=7
**V_5^+**	isomer A	0.215	**0.000**	0.196	0.568
	isomer B	0.466	0.524	1.307	\
**V_9^+**	isomer A	**0.000**	0.041	0.281	\
	isomer B	0.298	0.355	0.634	\
**Default Convergence Threshold**					
**Force Maximum**		0.000450			
**Force RMS**		0.000300			
**Displacement Maximum**		0.001800			
**Displacement RMS**		0.001200			

**V_n^+ Cluster**		**Relative Energies (eV)**			
		M=1	M=3	M=5	M=7
V_5^+	isomer A	0.246	**0.000**	0.107	0.568
	isomer B	0.465	0.524	1.307	\
V_9^+	isomer A	**0.000**	0.040	0.282	\
	isomer B	0.298	0.356	0.637	\
**Tight Convergence Threshold**					
Force Maximum		0.000015			
Force RMS		0.000010			
Displacement Maximum		0.000060			
Displacement RMS		0.000040			

Supplementary Table 4 Relative energies of $V_nH_2O^+$ (n=1-13) isomers calculated at BP86-D3/def2-TZVP level of theory. Energies are given in eV. Values of *M* refer to spin multiplicity.

$V_nH_2O^+$		Energies			
		M=1	M=3	M=5	M=7
**n=1**		3.29	1.00	**0.00**	5.42
**n=3**	a	0.52	**0.00**	0.31	
	b	0.52	0.11	0.40	
	c	0.53	0.32	0.40	
**n=5**	a	0.24	**0.00**	0.15	0.69
	b	0.35	0.15	0.21	0.71
**n=7**	a	**0.00**	0.18	0.43	
	b	0.15	0.28	0.53	
**n=9**	a	**0.00**	0.11	0.43	
	b	0.07	0.15	0.36	
	c	0.18	0.27	0.47	
	d	0.20	0.32	0.46	
**n=11**	a	**0.00**	0.04		
	b	0.03	0.002		
	c	0.11	0.14		
	d	0.14	0.16		
	e	0.15	0.18		
	f	0.20	0.24		
	g	0.34	0.42		
**n=13**	a	**0.00**	0.11		
	b	0.07	0.09		
	c	0.13	0.21		

$V_nH_2O^+$		Energies			
		M=2	M=4	M=6	M=8
**n=2**		**0.00**	0.12	1.35	
**n=4**	a	**0.00**	0.37	1.07	
	b	0.33	0.39	0.81	
**n=6**	a	0.07	**0.00**	0.67	
	b	0.51	0.01	0.99	
	c	0.31	0.52	1.06	
**n=8**	a	**0.00**	0.21	0.82	
	b	0.01	0.29	0.94	
	c	0.03	0.23	0.89	
	d	0.10	0.32	1.01	
**n=10**	**a**	**0.00**	0.47		
	b	0.04	0.47	1.24	
	c	0.11	0.64		
**n=12**	a	**0.00**	0.18		
	b	0.02	0.18		
	c	0.05	0.24		

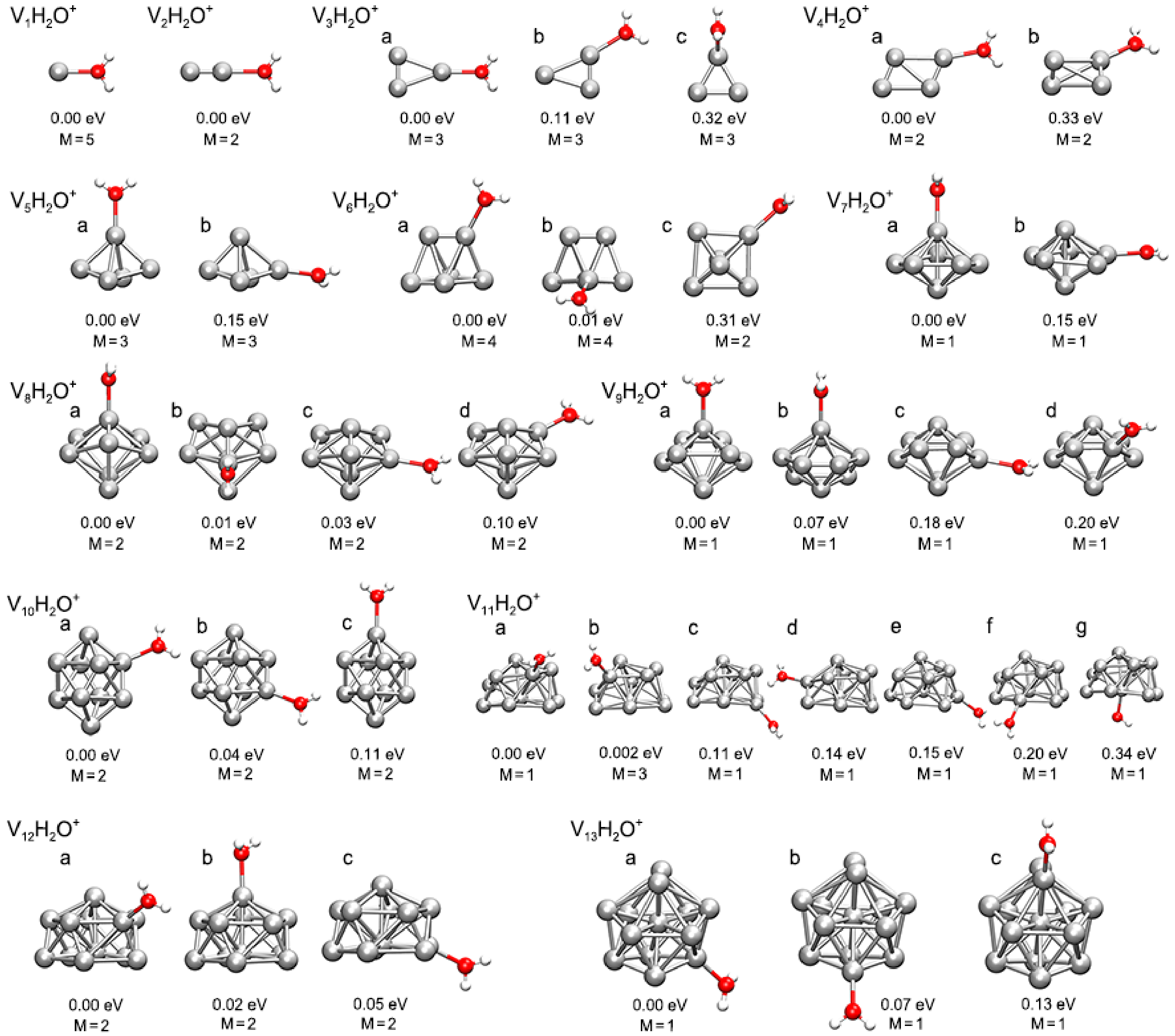

Supplementary Figure 6 Isomer structures and the relative energies (in eV) of $V_nH_2O^+$ (n=1-13) clusters calculated at BP86-D3/def-2TZVP level. M indicates spin multiplicity.

Supplementary Table 5 Relative energies of V_nO^+ isomers calculated at BP86-D3/def2-TZVP level of theory. Energies are given in eV. The values of M refer to spin multiplicity.

V_nO^+		Energies		
		M=1	M=3	M=5
**n=1**		1.09	**0.00**	3.57
**n=3**	a	**0.00**	0.42	0.86
	b	0.73	0.79	0.86
**n=5**	a	**0.00**	0.14	0.64
	b	0.07	0.49	0.62
**n=7**	a	**0.00**	0.25	0.63
	b	0.55	0.17	0.93
**n=9**	a	**0.00**	0.05	0.22
	b	0.15	0.17	0.47
	c	0.31	0.63	0.76
	d	0.39	0.32	0.56
	e	0.37	0.33	0.57
	f	0.47	0.62	0.65
**n=11**	a	**0.00**	0.26	
	b	0.13	0.20	
	c	0.16	0.27	
	d	0.23	0.17	
	e	0.21	0.33	
	f	0.22	0.31	
	g	0.33	0.36	
	h	0.47	0.33	
	i	0.47	0.45	
	j	0.48	0.47	
**n=13**	a	**0.00**	0.01	
	b	0.11	0.05	
	c	0.09	0.21	

V_nO^+		Energies		
		M=2	M=4	M=6
n=2	a	**0.00**	0.40	0.39
	b	0.26	0.40	0.39
n=4	a	**0.00**	1.04	1.27
	b	0.35	0.79	0.65
	c	0.62	1.10	0.65
n=6	a	**0.00**	0.44	0.68
	b	0.54	0.57	0.70
	c	0.61	0.83	1.17
	d	1.01	1.12	1.35
	e	1.07	1.15	1.58
	f	1.34	1.25	1.60
n=8	a	**0.00**	0.28	0.74
	b	0.38	0.50	0.71
	c	0.39	1.18	0.94
	d	0.75	1.11	1.69
	e	0.94	0.86	1.23
	f	0.95	1.23	1.30
	g	2.10	1.01	1.96
n=10	a	**0.00**	0.31	
	b	0.02	0.43	
	c	0.21	0.64	
	d	0.34	0.67	
n=12	a	**0.00**	0.18	
	b	0.06		
	c	0.13		
	d	0.18	0.36	
	e	0.32	0.46	
	f	0.38		

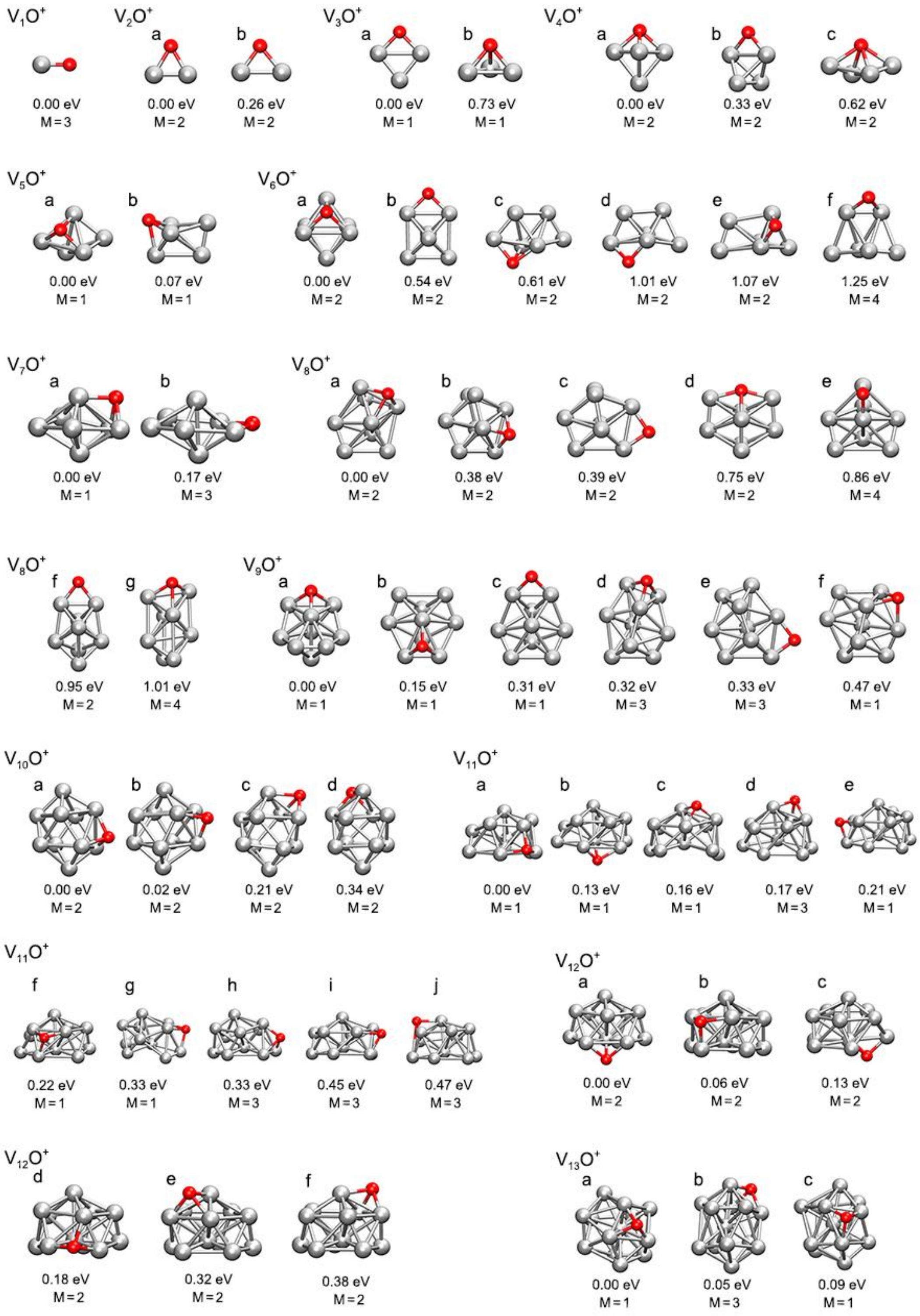

Supplementary Figure 7 The isomer structures and the relative energy difference (in eV) of V_nO^+ (n=1-13) clusters calculated at BP86-D3/def-2TZVP level. M indicates spin multiplicity.

Supplementary Table 6 Electronic states of the low-lying energy isomers of V_n^+, $V_nH_2O^+$, V_nO^+ clusters (n=1-13) calculated at BP86-D3/def-2TZVP level.

**Number of V Atoms**	**V_n^+ (symmetry)**	**$V_nH_2O^+$**	**V_nO^+**
**n=1**	^5D	$^5B_1$	$^3\Sigma$
**n=2**	$^4\Sigma_g$ ($D_{\infty h}$)	^2A	$^2A'$
**n=3**	$^3B_1$ (C_{2v})	$^3B_1$	$^1A'$
**n=4**	$^2A'$ (C_s)	^2A	^2A
**n=5**	^3A	^3A	^1A
**n=6**	^4A (C_2)	^4A	^2A
**n=7**	^1A	^1A	^3A
**n=8**	^2A	^2A	^2A
**n=9**	$^1A'$ (C_S)	^1A	^1A
**n=10**	$^2B_3$ (D_2)	^2A	^2A
**n=11**	^1A	^1A	^1A
**n=12**	^2A	^2A	^2A
**n=13**	^1A	^1A	^1A

S5 DFT-Calculated Vibration Modes

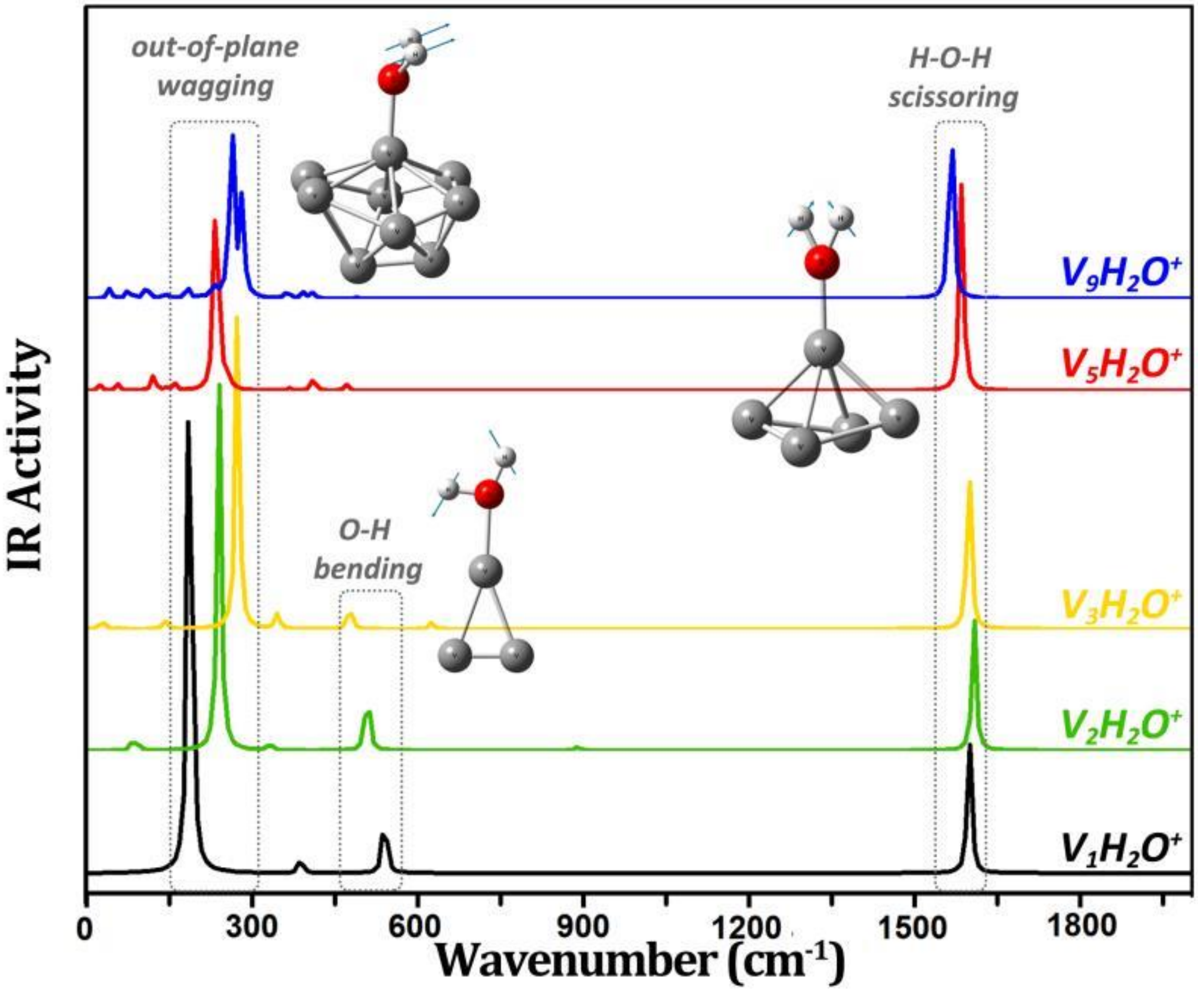

Supplementary Figure 8 The IR activities of the $V_nH_2O^+$ (n = 1, 2, 3, 5, 9) clusters at ground states calculated at BP86-D3/def2-TZVP level of theory. The frequency values are not corrected by any scale factor.

The calculated IR activities of the $V_nH_2O^+$ clusters show that there are O-H bending vibrations for $V_{1-3}H_2O^+$ at ~550 cm^{-1} which benefit the following formation of O-H-V transition state (Figure 4 of the main text). However, this O-H bending mode is almost negligible for $V_5H_2O^+$ and $V_9H_2O^+$ (which begin to display more and more weak low-frequency vibrations); instead, the out-of-plane wagging and H-O-H scissoring allow the subsequent steering of $-OH_2$ towards the formation of an O-VV-V transition state (Figure 5 of the main text).

S6 Energetics

Supplementary Table 7 The DFT-calculated V-atom removal energy, $V_n^+ \rightarrow V_{n-1}^+ + V$ (eV), with a comparison with the previous calculation results and experimental study.

V_n^+	BP86-D3 /def2TZVP (ZPVE)	BP86-D3 /def2TZVP (Gibbs free energy at 1 atm, 298 K)	Literature work[2] BPW91-D2 /6-311++G(2d,2p)	Experimental dissociation thresholds[3]	Other literature work
1	---	---	---	---	
2	4.69	4.41	3.54	3.143 (0.003)	
3	3.49	3.23	2.4	2.27 (0.09)	
4	4.65	4.23	3.32	3.53(0.08)	
5	4.07	3.72	2.83	3.24 (0.17)	
6	4.99	4.56	3.69	4.13 (0.16)	
7	4.95	4.52	3.58	3.86 (0.17)	
8	5.00	4.60	3.75	3.99 (0.17)	
9	4.74	4.32	3.22	3.67 (0.23)	
10	5.14	4.73	3.85	3.96 (0.21)	
11	4.75	4.35	3.16	3.96 (0.24)	
12	5.06	4.70	3.46	4.13 (0.29)	
13	5.26	4.86	3.73	4.65 (0.32)	4.35 ref.[4]<br>5.4 ref.[5]<br>5.3 ref.[6]

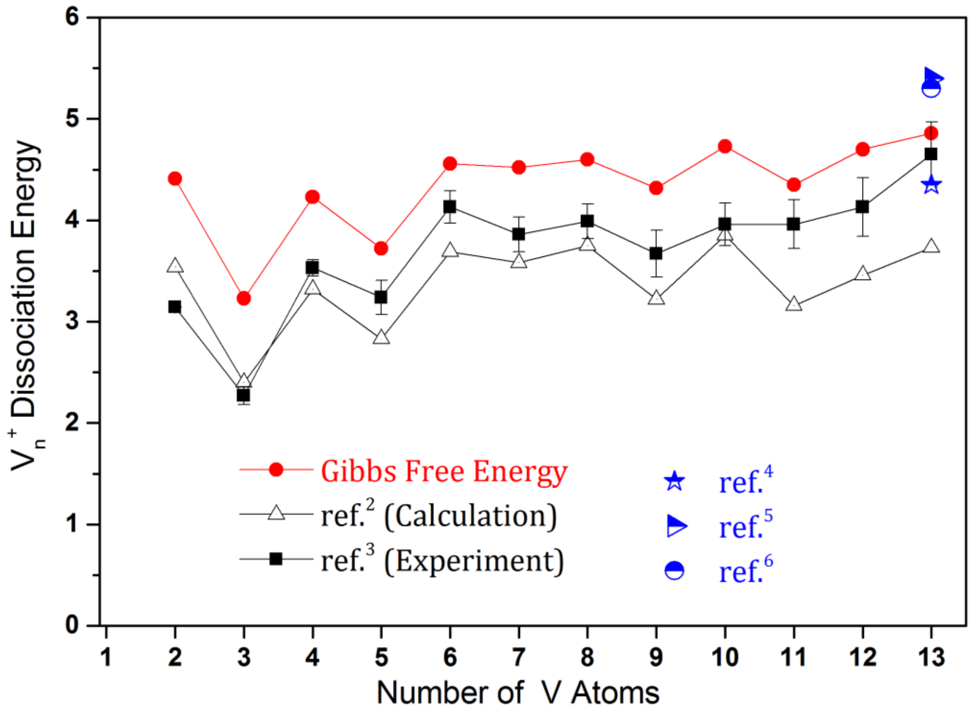

Supplementary Figure 9 DFT-calculated V-atom removal energy (given by Gibbs free energy at 298K), $V_n^+ \rightarrow V_{n-1}^+ + V$, with a comparison with the previous calculation results,[2] and experimental study.[3, 4, 5, 6]

In actual experiments, there is coexistence of cations and anions and electrons generated in the LaVa source. Thus, the subsequent cluster reaction products may undergo dissociation and neutralization processes in the collision cell. In view of this, we examined the adiabatic ionization energies (I.E.) of the neutral V_nH_2O clusters, as shown in Supplementary Figure 10, where the clusters $V_{1,2}H_2O^+$ show larger electron affinities than other sizes indicating likely neutralization under multiple collisions.

Supplementary Table 8 Relative energies of neutral V_nH_2O (n=1-13) isomers, calculated at BP86-D3/def2-TZVP level of theory. Energies are given in eV. Values of *M* refer to spin multiplicity.

V_nH_2O	Energy					V_nH_2O	Energy		
	M=2	M=4	M=6	M=8			M=1	M=3	M=5
**n=1**		0.311	0.000	5.364		**n=2**	0.344	0.000	0.868
**n=3**	0.000	0.071				**n=4**	0.060	0.000	0.373
**n=5**	0.000	0.093				**n=6**	0.081	0.000	0.247
**n=7**	0.000	0.391				**n=8**	0.000	0.009	
**n=9**	0.000	0.341				**n=10**	0.000	0.690	
**n=11**	0.003	0.000	0.379			**n=12**	0.000	0.118	
**n=13**	0.000	0.156							

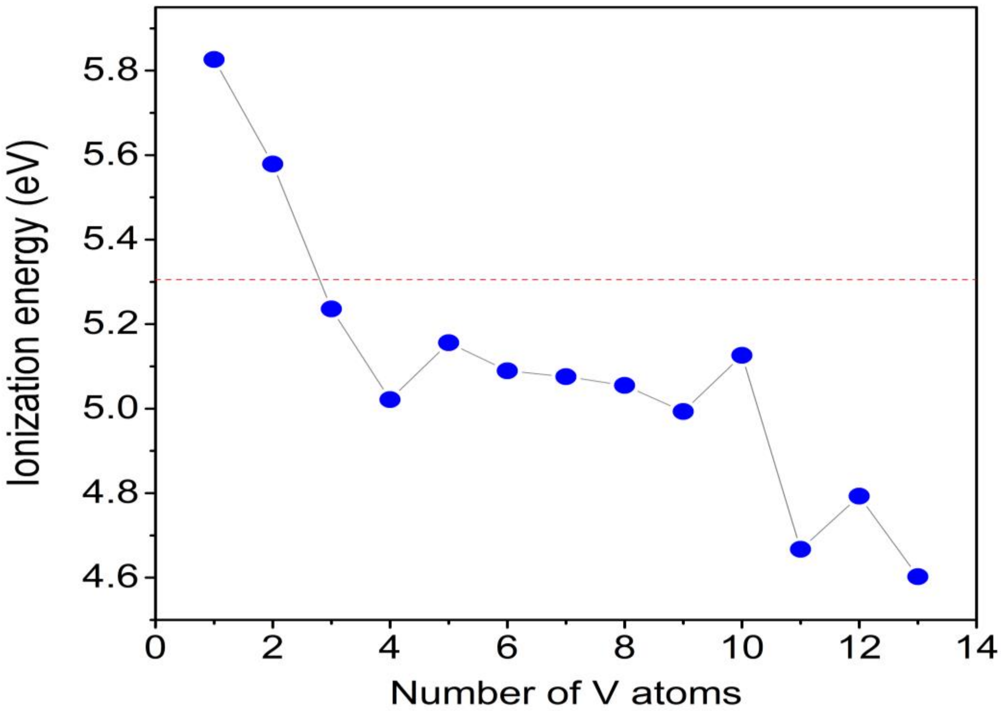

Supplementary Figure 10 Adiabatic ionization energies (I.E.) of neutral V_nH_2O clusters, indicating the varying electron affinities of the cationic $V_nH_2O^+$ clusters. The red dot line at IE = 5.3 eV is just drawn to guide the eye.

Supplementary Table 9 The DFT-calculated H_2O-binding energy, with a comparison with the previous calculation results and experimental study. Energies are given in eV.

V_n^+	BP86-D3 /def2TZVP (ZPVE)	BP86-D3 /def2TZVP (Gibbs free energy at 1 atm, 298 K)	BPW91-D2 /6-311++G(2d,2p)[2]	Experiment
1	1.860	1.612	1.643344	1.52 (0.05)[7]; 1.57 (0.13)[8]; 1.52 (0.17)[9]
2	1.349	1.023	1.166384	
3	1.279	0.923	1.005952	
4	1.140	0.796	0.971264	
5	1.282	0.921	0.93224	
6	1.213	0.835	1.023296	
7	1.321	0.949	1.092672	
8	1.224	0.856	0.992944	
9	1.324	0.961	1.092672	
10	1.173	0.805	0.923568	
11	1.190	0.817	1.005952	
12	1.151	0.778	0.884544	
13	1.197	0.795	0.927904	

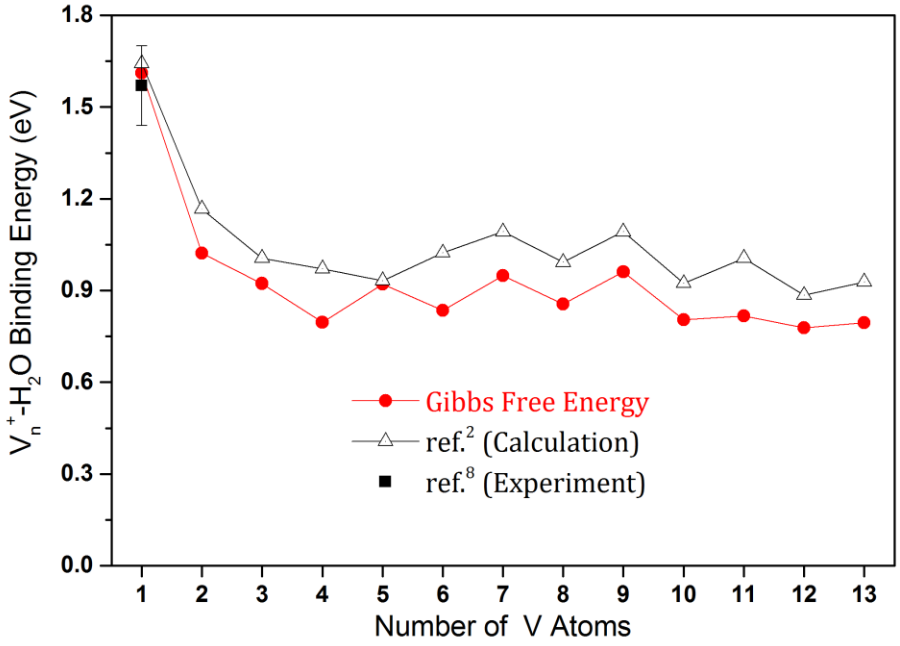

Supplementary Figure 11 A comparison of DFT-calculated H_2O-binding energy, with the previous calculation results and experimental study. The detailed values correspond to the above table.

Supplementary Table 10 The DFT-calculated O-bonding energy $E_{O-binding} = E(\mathrm{V}_n\mathrm{O}^+) - E(V_n{}^+) - E(O)$ on optimized ground-state V_nO^+ structures, and a comparison with the previous experimental dissociation threshold analysis. Energies are given in eV.

V_n^+	DFT calculations at BP86-D3/def2TZVP (ZPVE)	BP86-D3 /def2TZVP (Gibbs free energy at 1 atm, 298 K)	Experimental (ref.[10]) threshold analysis
1	*6.76*	6.50	*5.99 (0.10)*
2	*6.40*	6.11	*5.1 (0.3)*
3	*7.95*	7.51	*7.3 (0.3)*
4	*7.48*	7.10	*7.5 (0.3)*
5	*8.18*	7.72	*7.3 (0.4)*
6	*7.86*	7.47	*7.4 (0.7)*
7	*7.85*	7.22	*7.0 (0.7)*
8	*7.52*	7.12	*5.6 (0.4)*
9	*7.45*	7.05	*6.0 (0.8)*
10	*7.18*	6.78	*6.7 (0.4)*
11	*7.52*	7.11	*5.3 (0.4)*
12	*7.64*	7.20	*4.5 (0.6)*
13	*7.85*	7.42	*5.1 (0.5)*

Note: ZPVE, total energy corrected by zero-point vibrations; the Gibbs free energies correspond to 298K.

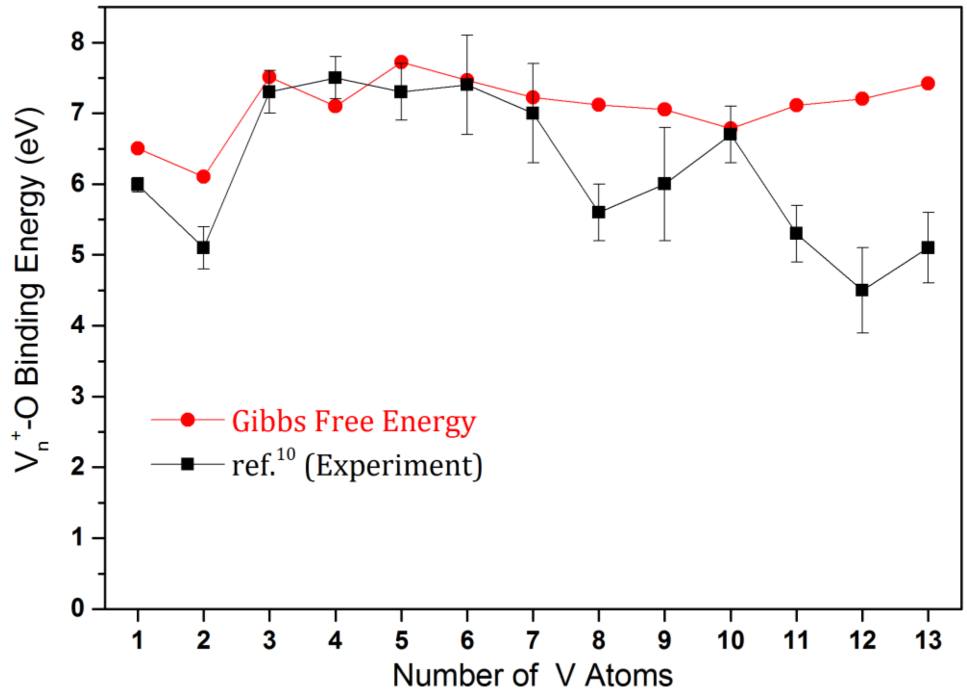

Supplementary Figure 12 A comparison of DFT-calculated O-binding energy, with a comparison with the previous experimental study.[10] The detailed values correspond to the above table.

By comparing the DFT-calculated O-binding energy with the previous experimental study on the bond energies of V_n^+-O by dissociation threshold analysis, there is a general consistence except a few clusters such as V_8^+ and V_{11-13}^+. The occasional inconsistence may be due to the following factors.

1) Vanadium clusters could radiate with a high recurrent fluorescence rate as established a long time ago,[5] while this radiative cooling effect was not considered by the mentioned literature in their data of the dissociation threshold analysis.[4, 11] One simple estimate suggests a 20% change in the value of the fitted dissociation energy,[4] and so display the error bars of the mentioned literature.[10]

2) Another effect that was not attracted sufficient attention is the thermal properties of metal clusters at high excitation energies. These are most likely not possible to describe by simple harmonic oscillator models. That is, the difference of the experimentally determined threshold values are likely associated with a variation of radiation-induced residual energy which could cause electronic excitation states or unthermalized geometric deformation /aggravated vibrations, as well as melting effect at reduced sizes.[12, 13, 14, 15, 16] Melting alone is enough to disprove the simplified harmonic oscillator hypothesis, such as that by the group of *Haberland* and *von Issendorff* on sodium clusters.[17] For vanadium clusters the consequences of this effect will most likely have an impact on numbers extracted from experiments but the magnitude is difficult to quantify before thermal properties of these clusters have been measured.

3) From the point of view of DFT calculations, a universally correct exchange and correlation functional is not yet available to the scientific community. Apart from that, the theoretical calculation method and basis set could also bring forth overall energy differences, although the size-dependent tendency is almost parallel.

S7 Natural Population Analysis of Charges

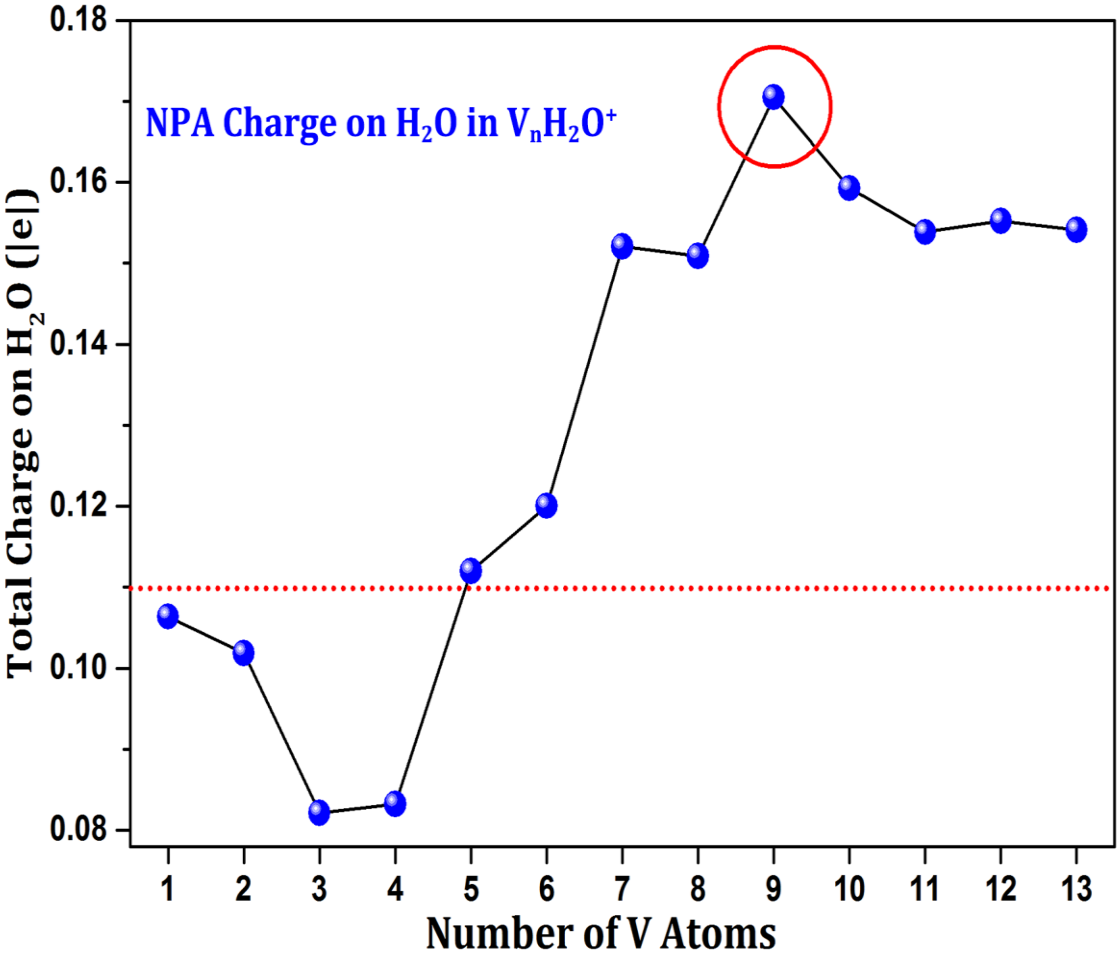

Supplementary Figure 13 Natural population analysis (NPA) charge on H_2O in $V_nH_2O^+$ clusters calculated at BP86-D3/def2-TZVP level using NBO6.0 method.[18] The red dot line at 0.11 is just drawn to guide the eye.

Supplementary Table 11 NPA charge distributions on the atoms of $V_nH_2O^+$ (n = 1-13) calculated at BP86-D3/def2-TZVP level using NBO6.0 method.[18] The vanadium atom adsorbing the water molecule is defined as V-1 and the other vanadium atoms connecting with V-1 are identified as V-2, V-3, V-4 and V-5, etc. Among them, the V-2 corresponds to the one with the largest positive partial charge, while V-3 aims at the minima.

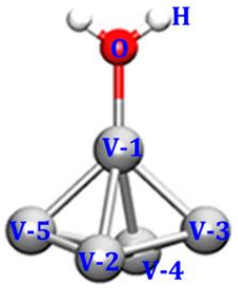

	H	O	V-1	V-2	V-3	V-4	V-5
**$V_1H_2O^+$**	0.53	-0.96	0.89	\	\	\	\
**$V_2H_2O^+$**	0.52	-0.94	0.39	0.51	\	\	\
**$V_3H_2O^+$**	0.52	-0.97	0.47	0.23	0.23	\	\
**$V_4H_2O^+$**	0.52	-0.95	0.44	0.08	0.04	0.08	\
**$V_5H_2O^+$**	0.53	-0.94	0.34	0.20	0.07	0.20	0.07
**$V_6H_2O^+$**	0.52	-0.92	0.09	0.14	0.10	0.14	0.19
**$V_7H_2O^+$**	0.52	-0.90	0.15	0.12	0.06	0.07	0.07
**$V_8H_2O^+$**	0.52	-0.89	-0.01	0.16	0.03	0.14	0.03
**$V_9H_2O^+$**	0.52	-0.87	-0.03	0.14	0.04	0.14	0.10
**$V_{10}H_2O^+$**	0.52	-0.88	-0.03	0.11	0.07	0.11	0.10
**$V_{11}H_2O^+$**	0.52	-0.88	-0.07	0.13	0.07	0.14	0.11
**$V_{12}H_2O^+$**	0.52	-0.88	-0.02	0.24	0.05	0.24	0.10
**$V_{13}H_2O^+$**	0.52	-0.88	0.01	0.19	0.06	0.21	0.13

S8 Frontier Orbitals

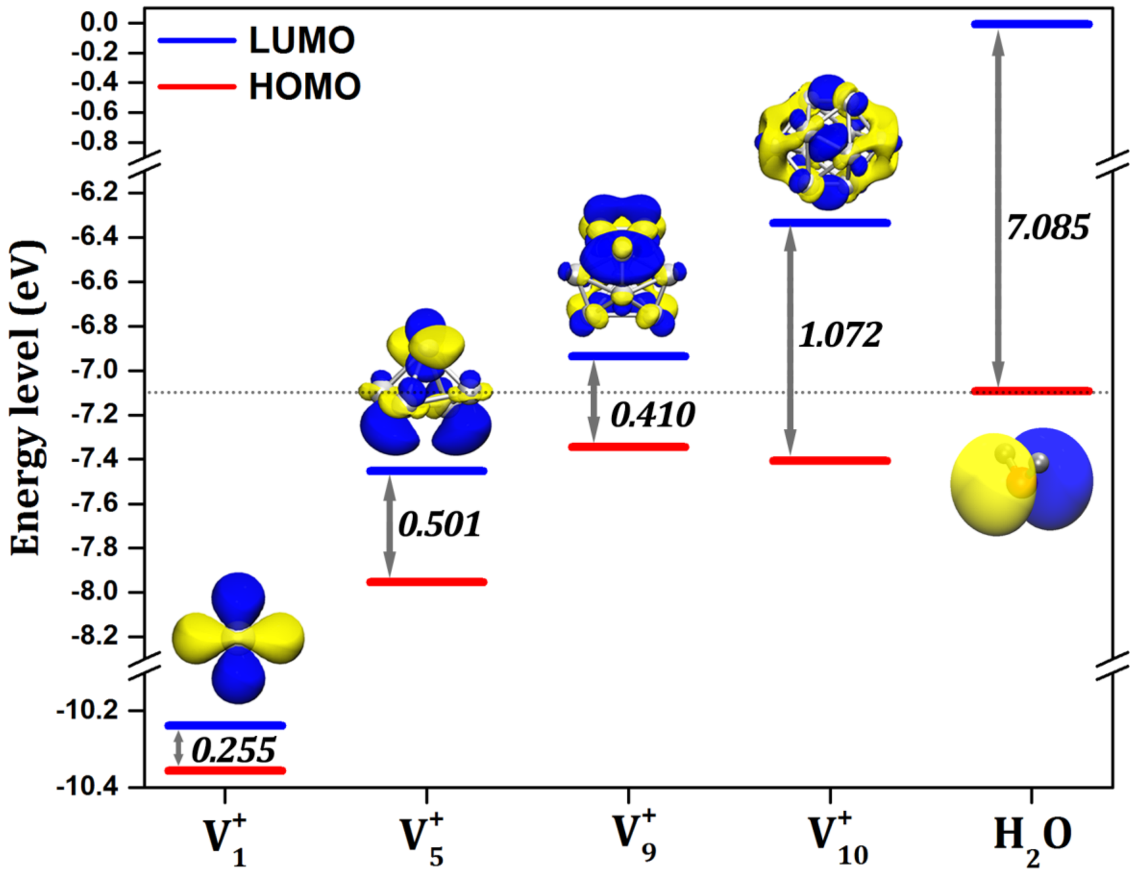

Supplementary Figure 14 The HOMO and LUMO energy levels of the chosen clusters V_1^+, V_5^+, V_9^+, V_{10}^+, with a comparison to that of H_2O. The dotted line at -7.1 eV corresponds to the HOMO energy level of H_2O.

The HOMO-LUMO gaps are often associated with the cluster stability and reaction inertness. Displayed here, are the HOMO and LUMO patterns of $V_{1,5,9,10}^+$ and H_2O, as well as their relative orbital energy levels. Considering that V_n^+ react with water by transfer of electron, the proximity of the HOMO of H_2O relative to the LUMO of $V_{5,9}$ facilitates electron transfer from oxygen lone pair of water to the vanadium cluster.

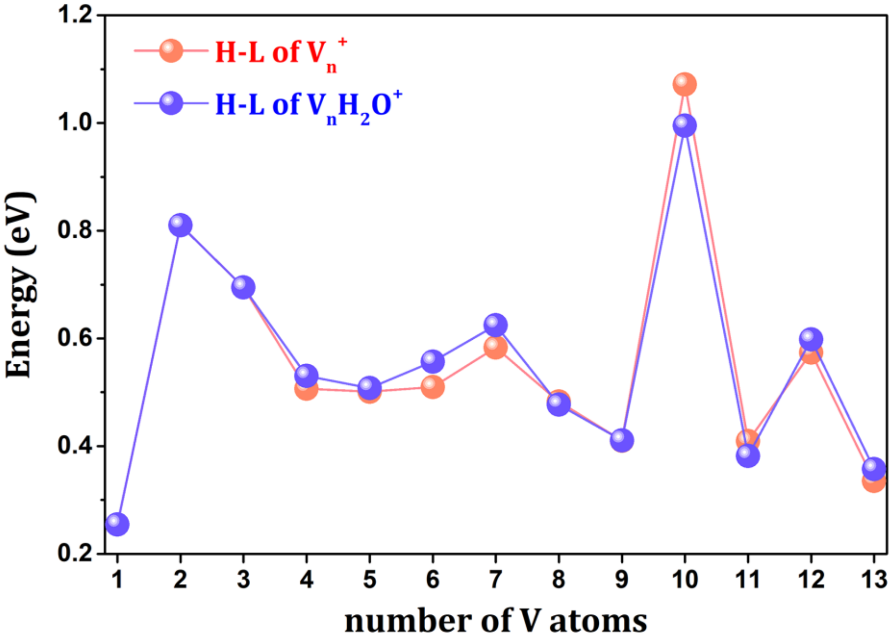

Supplementary Figure 15 HOMO-LUMO gaps of V_n^+ and $V_nH_2O^+$ clusters. Energies are given in eV.

S9 Energy Decomposition Analysis

Energy decomposition analysis (EDA) based on natural orbitals for chemical valence (NOCV) [19, 20, 21] was conducted using ADF software package. By taking V_n^+ and H_2O as interacting fragments, EDA-NOCV results for $V_nH_2O^+$ are calculated at the BP86-D3/TZ2P level using BP86-D3/ def2-TZVP optimized geometries. According to the general principle of EDA,[21, 22] the interaction energy (ΔE_{int}) between V_n^+ and H_2O in $V_nH_2O^+$ can be divided into three parts: $\Delta E_{int} = \Delta E_{pauli} + \Delta E_{elstat} + \Delta E_{orb}$, where ΔE_{pauli} is the repulsion energy caused by the Pauli exclusion principle, and ΔE_{elstat} and ΔE_{orb} are the attraction energies due to electrostatic and orbital interactions, respectively. As seen in Supplementary Figure 16 and Table 12 below, the contribution of ΔE_{elstat} to ΔE_{int} is larger than that of ΔE_{orb}; also, the plots of ΔE_{int} values show the same tendency of odd-even oscillations as that of the E_{ad} value plots (Figure 3a). From the ADF-calculated EDA results, the electrostatic interaction contributes about 2/3 to the attractive V_n^+-H_2O interaction, while the orbital interaction does ~1/3 contribution.

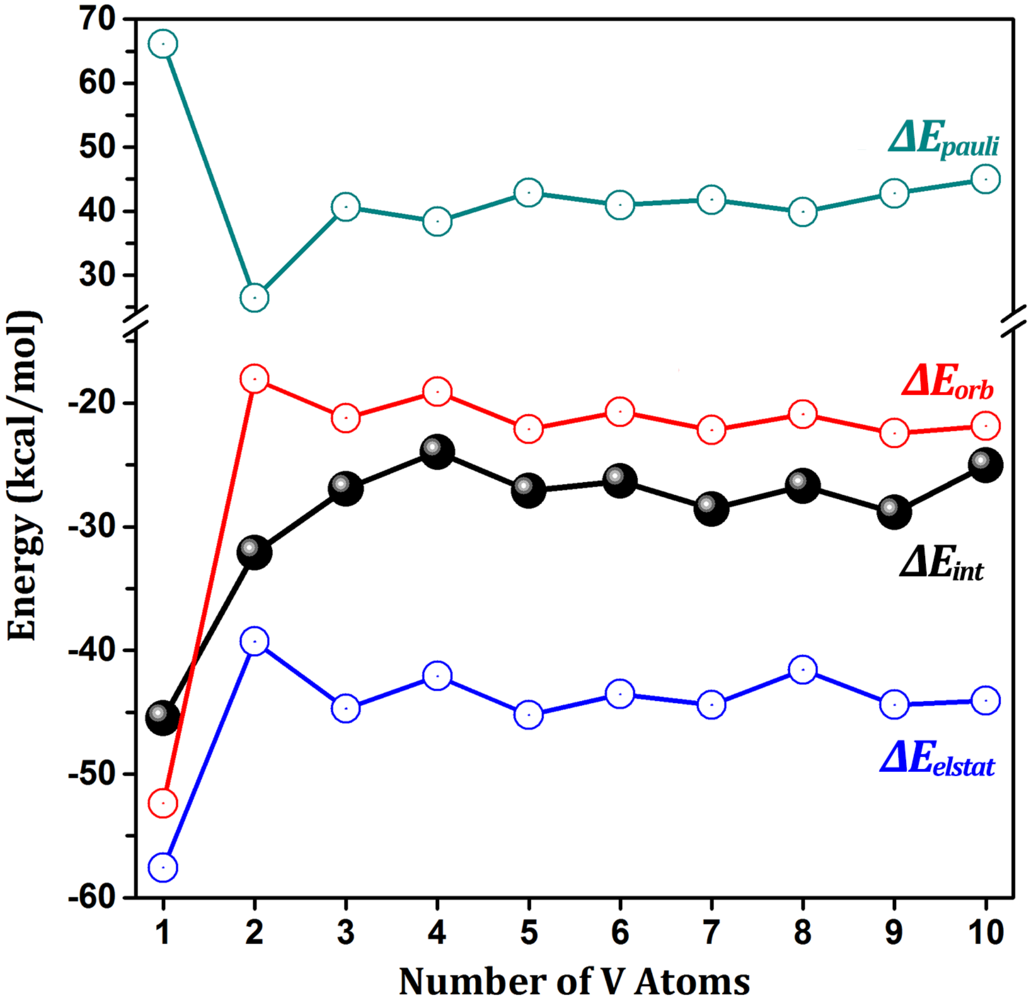

Supplementary Figure 16 Energy decomposition analysis for $V_nH_2O^+$ showing the contributions of electrostatic interactions and orbital interactions to the total bonding energies. Detailed energy values are given in Supplementary Table 12 below.

Supplementary Table 12 EDA-NOCV results for $V_nH_2O^+$ at the BP86-D3/TZ2P level using BP86-D3/ def2-TZVP optimized geometries, taking V_n^+ and H_2O as interacting fragments. Energy values are given in kcal/mol.

**Complex** (charge, α-β)	**Metal Cluster** (charge, α-β)	**Total Energy** ΔE_{tot}	**Electrostatic Interactio** ΔE_{elstat}	**Pauli Repulsion** ΔE_{pauli}	**Orbital Interactions** ΔE_{orb}	Converged?
V_1H_2O (1, 4)	(1, 4)	-45.48	-57.57	66.13	-52.37	yes
V_2H_2O (1, 1)	(1, 1)	-32.11	-39.28	26.44	-18.06	yes
V_3H_2O (1, 2)	(1, 2)	-26.94	-44.71	40.65	-21.23	yes
V_4H_2O (1, 1)	(1, 1)	-23.97	-42.08	38.36	-19.09	yes
V_5H_2O (1, 2)	(1, 2)	-27.11	-45.22	42.88	-22.12	yes
V_6H_2O (1, 3)	(1, 3)	-26.31	-43.54	40.94	-20.71	yes
V_7H_2O (1, 0)	(1, 0)	-28.58	-44.42	41.77	-22.19	yes
V_8H_2O (1, 1)	(1, 1)	-26.71	-41.58	39.86	-20.92	yes
V_9H_2O (1, 0)	(1, 0)	-28.84	-44.40	42.77	-22.44	yes
$V_{10}H_2O$ (1, 1)	(1, 1)	-25.04	-44.06	44.99	-21.86	yes
$V_{11}H_2O$ (1, 0)	(1, 0)	-12001.32	-115.41	50.26	-11931.98	no
$V_{12}H_2O$ (1, 1)	(1, 1)	-7949.85	-115.61	173.81	-8003.99	moderately
$V_{13}H_2O$ (1, 0)	(1, 0)	7332.57	-48.34	49.85	7335.27	no

(charge, α-β): (number net charge, number of spinα-spinβ electrons)

$\Delta E_{tot} = \Delta E_{pauli} + \Delta E_{elstat} + \Delta E_{orb}$

S10 Reaction Coordinate of "V_3^+ + $2H_2O$"

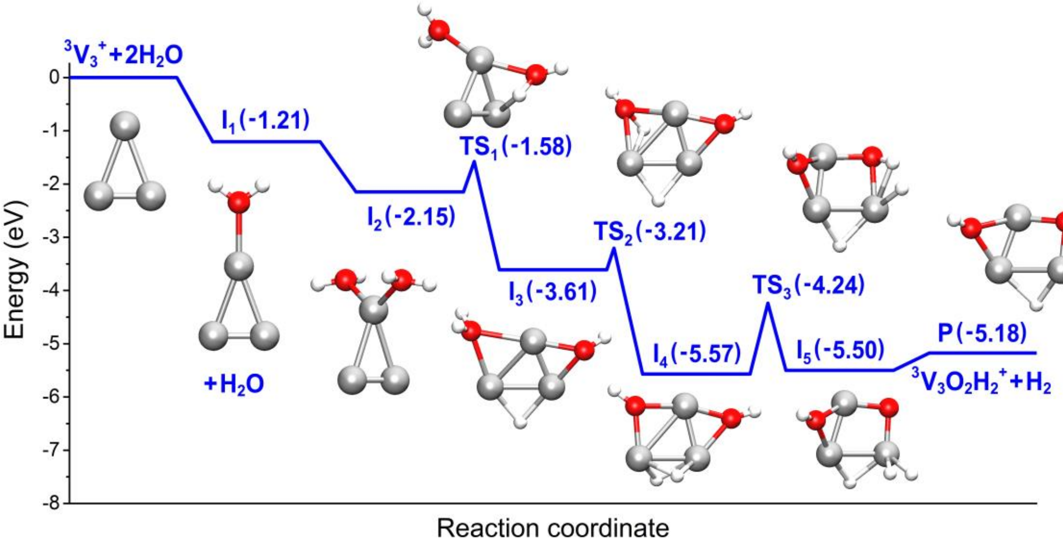

Supplementary Figure 17 Reaction coordinate of "$V_3^+ + 2H_2O \rightarrow V_3O_2H_2^+ + H_2$". The energy values are relative to the entrance channel, calculated at BP86 /def2-TZVP level of theory.

Supplementary References

1. Cheng P, Koyanagi GK, Bohme DK. Heavy Water Reactions with Atomic Transition-Metal and Main-Group Cations: Gas Phase Room-Temperature Kinetics and Periodicities in Reactivity. *J Phys Chem A* **111**, 8561-8573 (2007).
2. Meza B, Miranda P, Castro M. Structural and Electronic Properties of Hydrated V_nH_2O and $V_n^+H_2O$, n ≤13, Systems. *J Phys Chem C* **121**, 4635-4649 (2017).
3. Su CX, Hales DA, Armentrout PB. Collision-Induced Dissociation of V_n^+ (N=2-20) with Xe - Bond-Energies, Dissociation Pathways, and Structures. *J Chem Phys* **99**, 6613-6623 (1993).
4. Hansen K, Herlert A, Schweikhard L, Vogel M, Walther C. The dissociation energy of V_{13}^+ and the consequences for radiative cooling. *Eur Phys J D* **34**, 67-71 (2005).
5. Walther C*, et al.* Radiative Cooling of a Small Metal Cluster: The Case of V_{13}^+. *Phys Rev Lett* **83**, 3816-3819 (1999).
6. Margrave JL. *Vapour pressure of the chemical elements (Nesmeyanov, A. N.)*. American Chemical Society (1964).
7. Dalleska NF, Honma K, Sunderlin LS, Armentrout PB. Solvation of transition-metal ions by water - sequential binding-energies of $M^+(H_2O)_x$, (x=1-4) for m=Ti to Cu determined by collision-induced dissociation. *J Am Chem Soc* **116**, 3519-3528 (1994).
8. Magnera TF, David DE, Michl J. GAS-PHASE WATER AND HYDROXYL BINDING-ENERGIES FOR MONOPOSITIVE 1ST ROW TRANSITION-METAL IONS. *J Am Chem Soc* **111**, 4100-4101 (1989).

9. Marinelli PJ, Squires RR. SEQUENTIAL SOLVATION OF ATOMIC TRANSITION-METAL IONS - THE 2ND SOLVENT MOLECULE CAN BIND MORE STRONGLY THAN THE 1ST. *J Am Chem Soc* **111**, 4101-4103 (1989).
10. Xu J, Rodgers MT, Griffin JB, Armentrout PB. Guided Ion Beam Studies of the Reactions of V_n^+ (n=2-17) with O_2: Bond Energies and Dissociation Pathways. *J Chem Phys* **108**, 9339-9350 (1998).
11. Vogel M, Hansen K, Herlert A, Schweikhard L. Model-free determination of dissociation energies of polyatomic systems. *Phys Rev Lett* **87**, 013401 (2001).
12. Martin TP, Näher U, Schaber H, Zimmermann U. Evidence for a size - dependent melting of sodium clusters. *J Chem Phys* **100**, 2322-2324 (1994).
13. Errandonea D, Boehler R, Ross M. Melting of the rare earth metals and f-electron delocalization. *Phys Rev Lett* **85**, 3444-3447 (2000).
14. Cao B, Starace AK, Judd OH, Jarrold MF. Phase coexistence in melting aluminum clusters. *J Chem Phys* **130**, 204303 (2009).
15. Cao B, Starace AK, Judd OH, Jarrold MF. Melting dramatically enhances the reactivity of aluminum nanoclusters. *J Am Chem Soc* **131**, 2446-2447 (2009).
16. Rapacioli M, Tarrat N, Spiegelman F. Melting of the Au_{20} Gold Cluster: Does Charge Matter? *J Phys Chem A*, (2018).
17. Hock C*, et al.* Premelting and postmelting in clusters. *Phys Rev Lett* **102**, 043401 (2009).
18. Glendening ED*, et al.* NBO 6.0. (ed^(eds) (2013).
19. Michalak A, Mitoraj M, Ziegler T. Bond Orbitals from Chemical Valence Theory. *J Phys Chem A* **112**, 1933-1939 (2008).
20. Mitoraj M, Michalak A. Natural Orbitals for Chemical Valence as Descriptors of Chemical Bonding in Transition Metal Complexes. *Journal of molecular modeling* **13**, 347-355 (2007).
21. Mitoraj MP, Michalak A, Ziegler T. A Combined Charge and Energy Decomposition Scheme for Bond Analysis. *J Chem Theory Comput* **5**, 962-975 (2009).
22. Hopffgarten Mv, Frenking G. Energy Decomposition Analysis. *WIREs Comput Mol Sci* **2**, 43-62 (2012).

23. Zhu J*, et al.* Boundary activated hydrogen evolution reaction on monolayer MoS_2. *Nat commun* **10**, 1348 (2019).
24. Wang P*, et al.* A broadband and strong visible-light-absorbing photosensitizer boosts hydrogen evolution. *Nat commun* **10**, 3155 (2019).
25. Schmider HL, Becke AD. Chemical content of the kinetic energy density. *Journal of Molecular Structure: THEOCHEM* **527**, 51-61 (2000).
26. Dotan H*, et al.* Decoupled hydrogen and oxygen evolution by a two-step electrochemical–chemical cycle for efficient overall water splitting. *Nature Energy* **4**, 786-795 (2019).
27. Cui C, Luo Z, Yao J. Enhanced Catalysis of Pt_3 Clusters Supported on Graphene for N–H Bond Dissociation. *CCS Chemistry* **1**, 215-225 (2019).
28. Vyboishchikov SF, Sauer J. Gas-phase vanadium oxide anions: Structure and detachment energies from density functional calculations. *J Phys Chem A* **104**, 10913-10922 (2000).
29. Li D*, et al.* Highly Quaternized Polystyrene Ionomers for High Performance Anion Exchange Membrane Water Electrolysers. *Nature Energy* **5**, 378-385 (2020).
30. Tian Lu, Chen F. Multiwfn: A Multifunctional Wavefunction Analyzer. *J Comput Chem* **33**, 580-592 (2012).
31. Li Y, Wei X, Chen L, Shi J, He M. Nickel-molybdenum nitride nanoplate electrocatalysts for concurrent electrolytic hydrogen and formate productions. *Nat commun* **10**, 5335 (2019).

32. Jensen KP, Roos BO, Ryde U. Performance of density functionals for first row transition metal systems. *J Chem Phys* **126**, 014103 (2007).
33. Scharfschwerdt B, van der Linde C, Petru Balaj O, Herber I, Schütze D, Beyer MK. Photodissociation and Photochemistry of $V^+(H_2O)_n$, n = 1–4, in the 360–680 nm Region. *J Low Temp Phys* **38**, 717-722 (2012).
34. VandeVondele J, Krack M, Mohamed F, Parrinello M, Chassaing T, Hutter J. Quickstep: Fast and Accurate Density Functional Calculations Using a Mixed Gaussian and Plane waves Approach. *Comput Phys Commun* **167**, 103-128 (2005).
35. Luo Z*, et al.* Reactant friendly hydrogen evolution interface based on di-anionic MoS_2 surface. *Nat commun* **11**, 1116 (2020).
36. Kweon DH*, et al.* Ruthenium anchored on carbon nanotube electrocatalyst for hydrogen production with enhanced Faradaic efficiency. *Nat commun* **11**, 1278 (2020).
37. Ditchfield R. Self-Consistent Perturbation Theory of Diamagnetism. *Mol Phys* **27**, 789-807 (1974).
38. Zhang P*, et al.* Streamlined hydrogen production from biomass. *Nature Catalysis* **1**, 332-338 (2018).
39. Kleinpeter E, Klod S, Koch A. Visualization of Through Space NMR Shieldings of Aromatic and Anti-Aromatic Molecules and a Simple Means to Compare and Estimate Aromaticity. *J Mol Struct: THEOCHEM* **811**, 45-60 (2007).
